# Discovery of novel genic-SSR markers from transcriptome dataset of an important non-human primate, *Macaca fascicularis*

**DOI:** 10.1038/s41598-019-44870-4

**Published:** 2019-06-11

**Authors:** Wendy Chang, J. EE-ULI, W. L. NG, Jeffrine J. Rovie-Ryan, S. G. Tan, Christina S. Y. Yong

**Affiliations:** 1 Department of Biology, Faculty of Science, Universiti Putra Malaysia, 43400 Serdang, Selangor Malaysia; 2 Department of Cell and Molecular Biology, Faculty of Biotechnology and Biomolecular Sciences, Universiti Putra Malaysia, 43400 Serdang, Selangor Malaysia; 3 China-ASEAN Institute of Marine Sciences, Xiamen University Malaysia, Jalan Sunsuria, Bandar Sunsuria, 43900 Sepang, Selangor Darul Ehsan Malaysia; 4 National Wildlife Forensic Laboratory (NWFL), Department of Wildlife and National Parks, KM 10, Jalan Cheras, 56100 Kuala Lumpur, Malaysia

**Keywords:** Population genetics, Genetic variation, Transcriptomics

## Abstract

*Macaca fascicularis*, also known as the cynomolgus macaque, is an important non-human primate animal model used in biomedical research. It is an Old-World primate widely distributed in Southeast Asia and is one of the most abundant macaque species in Malaysia. However, the genetic structure of wild cynomolgus macaque populations in Malaysia has not been thoroughly elucidated. In this study, we developed genic-simple sequence repeat (genic-SSR) markers from an in-house transcriptome dataset generated from the Malaysian cynomolgus macaque via RNA sequencing, and applied these markers on 26 cynomolgus macaque individuals. A collection of 14,751 genic-SSRs were identified, where 13,709 were perfect SSRs. Dinucleotide repeats were the most common repeat motifs with a frequency of 65.05%, followed by trinucleotide repeats (20.55%). Subsequently, we designed 300 pairs of primers based on perfect di- and trinucleotide SSRs, in which 105 SSRs were associated with functional genes. A subset of 30 SSR markers were randomly selected and validated, yielding 19 polymorphic markers with an average polymorphism information content value of 0.431. The development of genic-SSR markers in this study is indeed timely to provide useful markers for functional and population genetic studies of the cynomolgus macaque and other related non-human primate species.

## Introduction

*Macaca fascicularis* (Raffles 1821), also known as the ‘long-tailed macaque’ or the ‘cynomolgus macaque’, is a macaque species that is native to Southeast Asia and widely distributed in Malaysia, Thailand, Myanmar, Laos, Cambodia, Vietnam, Indonesia, Timor Leste, and the Philippines^1^. Despite being one of the predominant macaque species in Malaysia, the genetic structure of their wild populations remains unclear. In Malaysia, most studies were conducted to examine the distribution^2^, behaviour^3^, human-macaque conflict^4,5^ and their association with zoonotic diseases^6,7^. Only a few genetic studies involving phylogeography and population genetics of cynomolgus macaques were conducted thus far. Most genetic studies conducted were based on the maternally inherited mtDNA marker^8–10^, and a few reports were based on the Y-chromosome^11^ and genomic SSR markers^12,13^.

Simple sequence repeats (SSRs) are repetitive DNA sequences, generally with motifs of 2–6 bp long, and present abundantly in eukaryotic genome. Its codominant and multi-allelic properties are highly valued by geneticist and evolutionary biologist, and are commonly used as DNA markers in genetic studies. Despite the recent thriving of single nucleotide polymorphism (SNP) markers, SSR markers are still relevant in many applications^14–18^. SSRs can be broadly categorized into genomic SSR and genic-SSR, depending on their locations in the genome. SSRs sited in the transcribed region are generally known as genic-SSRs. As more SSRs associated with protein coding genes are found, it is more evident now that the previously presumed junk-DNA possibly play a crucial role in adaptive evolution^19^. While genic-SSR is not as abundant and as polymorphic as genomic SSR, it offers several advantages over genomic SSR marker – higher probability of finding association with functional gene, higher degree of transferability across related species, and lower occurrence of null alleles^20,21^. Despite its lower polymorphism level compared to genomic SSR, genic-SSR has been used successfully in population genetic and evolutionary studies in many species^20–23^

In recent years, advancement in sequencing technologies has made whole-genome or transcriptome sequencing of both model and non-model organisms feasible. The massive amount of transcriptome data obtained via RNA sequencing can be used in various applications, from gene identification to comparative functional analysis and differential gene expression. It also serves as an excellent sequence resource for marker development. Transcriptome sequencing coupled with established bioinformatic pipeline have been used effectively for high throughput identification of genic-SSR markers from various organisms^24–26^. Some of the tools used for SSR mining include MicroSatellite identification tool (MISA)^27^, FullSSR^28^ and Genome-wide Microsatellite analysing tool package (GMATA)^29^.

There are fewer reported studies on the development of SSR markers for the cynomolgus macaque compared to *Macaca mulatta*, another non-human primate model. Hitherto, development of genic-SSR markers from whole transcriptome sequencing data of cynomolgus macaque has yet to be attempted. Therefore, the present study aimed to develop genic-SSR markers from an in-house transcriptome dataset of the Malaysian cynomolgus macaque generated from our previous studies^30,31^. This study is the first comprehensive report on the development of genic-SSR markers from the transcriptome of cynomolgus macaque. Here, we mined sequences containing SSRs from the transcriptome dataset, designed primers flanking pure di- and trinucleotide SSRs, and identified their associations with functional genes. Some randomly selected markers were further validated. The genic-SSR markers reported in this study are useful for population, functional genomic and comparative mapping studies of cynomolgus macaque and other related species.

## Results and Discussion

### *De novo* assembly and functional annotation

*De novo* assembly of the transcriptome data generated a total of 597,457 contigs with an average contig length of 400 bp; minimum and maximum contig lengths of 178 bp and 21,411 bp, respectively. Of the total contigs generated, 356,560 (~60%) of the contigs had an average coverage of more than 10 reads, and annotation of these contigs revealed 73,880 (~21%) contigs associated with functional genes. Out of the 73,880 annotated contigs, 67,399 contigs matched to *M*. *fascicularis* (GCF 000364345.1) RNA sequences. Subsequent protein sequence similarity searched against *M*. *mulatta* (GCF 000772875.2), *Homo sapiens* (GRCH38) and SwissProt databases, further annotated 1,461, 742 and 4,278 contigs respectively.

### Identification and classification of genic-SSRs

**Figure 1:**
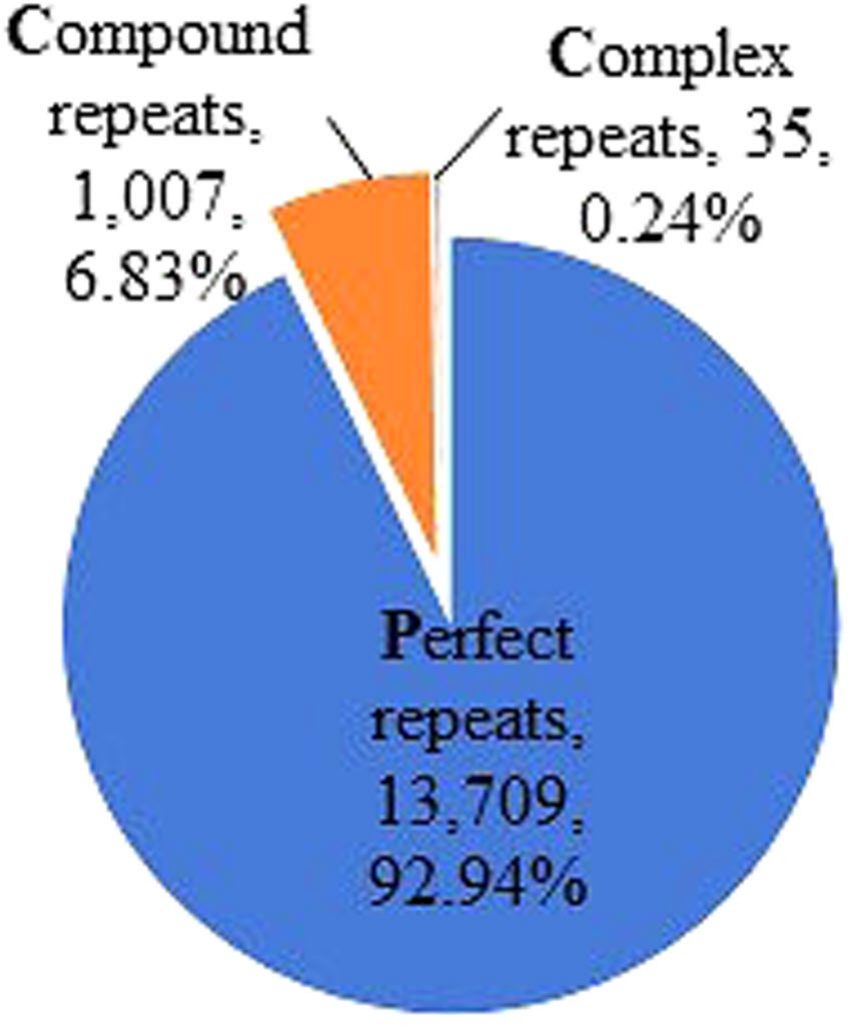
Classification of SSR types identified from the transcriptome sequences of *M*. *fascicularis*. SSR repeats were categorized into three groups: perfect, compound and complex SSRs.

We identified a total of 14,751 genic-SSRs in this study, reflecting the effectiveness of SSR mining from the transcriptome dataset. Of the total identified genic-SSRs, 13,709 (92.94%) were perfect repeats; while complex and compound repeats constituted the remaining 7.07% (Fig. 1). Among the perfect SSRs, dinucleotide repeats were the most abundant (8,918; 65.05%), followed by tri- (2,817; 20.55%), tetra- (1,062; 7.75%), penta- (767; 5.59%) and hexa- (145; 1.06%) nucleotide repeats. Di- and trinucleotide repeats constituted the largest groups of repeat motifs in our dataset, concurring with the results reported in other animal species such as human^32^, chicken^33^ and fish^34^.

**Figure 2:**
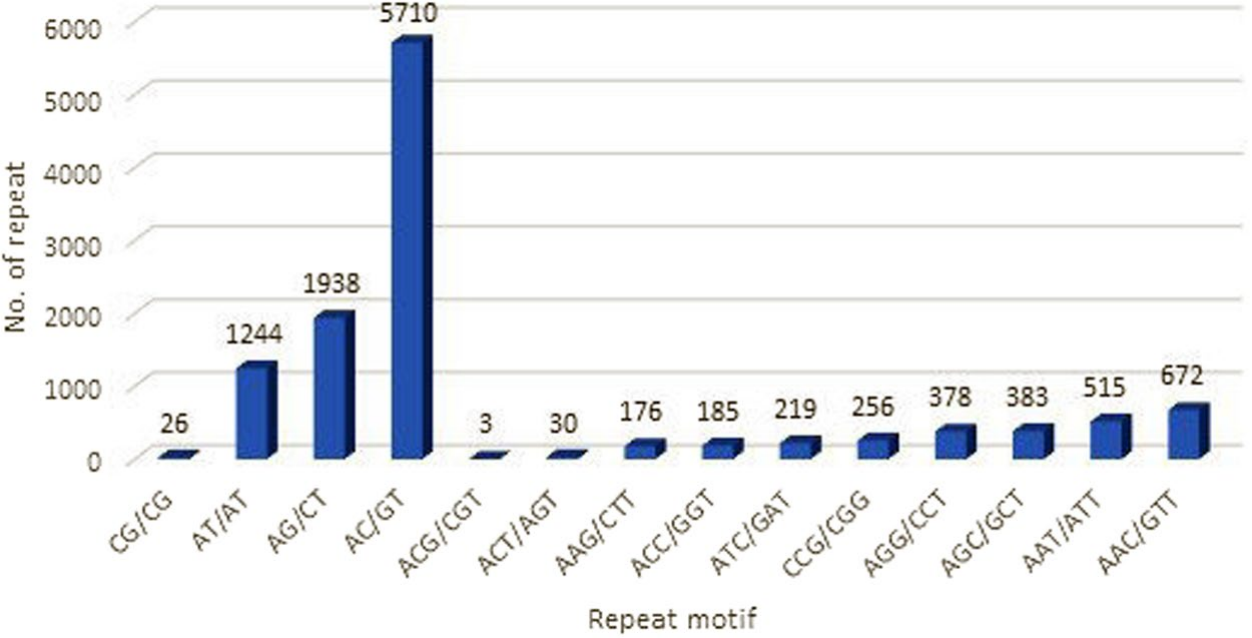
Frequency of di- and trinucleotide repeat motifs in the transcriptome of *M*. *fascicularis*.

Among the dinucleotide repeats, AC/GT (64.03%) accounted for the highest proportion, while CG/CG repeats were the lowest in proportion (0.29%). Amongst the ten types of trinucleotide repeats identified, AAC/GTT repeats were the most abundant (23.86%), and ACG/CGT repeats were the least common (~0.1%). The distributions of di- and trinucleotide SSRs according to motif are shown in Fig. 2. As for tetra-, penta- and hexanucleotide repeats, the most common motifs were AAAC/GTTT (8.3%), AAAAC/GTTTT (15.5%) and AAAAAC/GTTTTT (8.3%) respectively. Analysis of SSR densities in the human genome revealed that dinucleotide (AC/GT and AT/AT) and trinucleotide (AAC/GTT, AAT/ATT, AAG/CTT and AGG/CCT) repeats were the most common in humans^32^. AC/GT repeats were also reported to be the most common dinucleotide repeat in other organisms, including fish^35^ and sheep^36^. CG-rich SSR motifs are very rare in the transcriptome of the *M*. *fascicularis*, occurring less than 1%, which corroborated the results reported in the genomes of humans^32^ and other primate species^37^. CG/CG dinucleotide repeats are significantly low in vertebrates due to the methylation of cytosine, which favours the deamination of cytosine to thymidine^38^.

### Functional annotation of SSR loci, primer development and screening

Out of the 300 SSR loci used for primer design in this study, 105 loci were associated with genes involved in specific biological processes, cellular component and/or molecular functions. The complete list of these 300 SSRs and their respective predicted functions is provided in Supplementary Table S1. From the 30 SSR markers tested, 20 markers (66.67%) produced clear amplicons of expected sizes across all samples reproducibly. Nineteen out of these 20 markers (Table 1) were polymorphic, demonstrating that more than 60% (19 out of 30) of the markers screened in this study were polymorphic. Alignment of the sequences obtained from the PCR amplicons with the contig sequences used to design the primers also verified successful amplification of the targeted DNA regions.

**Table 1:** Twenty validated genic-SSR markers for *M*. *fascicularis*.

SSR locus	Amplicon size range (bp); number of repeats	Motif and repeat number based on transcriptome data	Motif observed based on sequenced PCR product	Polymorphic
MF016	350–375; 23–35	(GT)_16_	(GT) _n_	Yes
MF017	410–430; 17–27	(TC)_12_	(TC)_n_	No
MF021	435–450; 26–33	(TG)_13_	(TG)_n_	Yes
MF029	350–385; 18–35	(GT)_13_	(GT)_n_	Yes
MF069	400–425;9–21	(CA)_12_	(CA)_n_	Yes
MF080	285–315; 9–24	(TG)_13_	(TG)_n_	Yes
MF102	465–525; 6–36	(CA)_13_	(CA)_n_	Yes
MF113	400–450; 10–31	(CA)_17_	(CA)_n_	Yes
MF121	475–525; 15–38	(TG)_15_	(TG)_n_	Yes
MF130	440–450; 7–12	(GT)_10_	(GT)_n_	Yes
MF147	350–415; 7–39	(AC)_12_	(AC)_n_	Yes
MF188	445–460; 5–15	(AC)_11_	(AC)_n_	Yes
MF197	315–375; 6–35	(AT)_10_	(AT)_n_	Yes
MF225	325–375; 5–30	(AC)_10_	(AC)_n_	Yes
MF242	500–525; 18–30	(TG)_14_	(TG)_n_	Yes
MF255	335–400; 4–37	(GT)_12_	(GT)_n_	Yes
MF259	315–335; 8–18	(CA)_10_	(CA)_n_	Yes
MF261	265–285; 14–24	(CT)_18_	(CT)_n_	Yes
MF272	365–385; 5–14	(TG)_10_	(TG)_n_	Yes
MF273	435–450; 8–15	(TG)_11_	(TG)_n_	Yes

### Data analysis

Genetic diversity assessment was performed based on 19 polymorphic markers (Table 1) amplified across 26 *M*. *fascicularis* individuals, which were divided into the West Coast and East Coast populations. Heterozygosity assessment was performed on individual population (Supplementary Table S2), with West Coast and East Coast populations showed similar mean *H*_E_ values of 0.481 and 0.484, respectively. The mean *N*_A_ values for the West Coast population was 3.316 and East Coast population was 2.684. For overall genetic diversity assessment in all 26 individuals, *N*_A_, *H*_O_, *H*_E_, and PIC ranged from 2 to 6, 0 to 1, 0.125 to 0.713, and 0.110 to 0.653, respectively (Supplementary Table S3). The overall mean *N*_A_, *H*_O_, *H*_E_, and PIC were 3.630, 0.269, 0.495, and 0.431, respectively. *F*-statistics calculated from the 19 polymorphic loci revealed a mean *F*_ST_ of 0.059. Out of the 19 loci, three loci (MF121, MF259, and MF272) were the most polymorphic with six alleles each. Seven of the 19 polymorphic SSR loci had PIC values of >0.5, and thus, they were considered as highly informative^39^. Compared to previous population studies^12,13^, where genomic SSR markers were used, the genic-SSR markers used in this study generated lower *N*_A_, *H*_O_, *H*_E_ and PIC values for the same species. As the SSR markers developed in our study were generated from transcriptome, it was anticipated that the genetic diversity of these markers would be lower than those of SSR markers derived from genomic DNA regions^40^. The lower values could also be contributed by the lower number of samples (n = 26) and sampling sites in the current work compared to those studies^12,13^. Nonetheless, the identification of 19 polymorphic SSRs out of 30 markers screened based on 26 individual samples is promising. We are confident that higher *N*_A_, *H*_O_, *H*_E_ and PIC values would be obtained with more samples.

The average PIC value of 0.431 for the 19 polymorphic loci validated in this study was comparable to those genic-SSRs developed for the Korean quail (mean PIC value = 0.494)^26^ and crab (mean PIC value = 0.49)^24^. Although not all the 19 genic-SSR markers showed high polymorphism and PIC values, all showed the reproducibility and specificity highly desired in genotyping by PCR.

There were very few reported studies on the development of SSR markers for cynomolgus macaque. The first study conducted to develop SSR markers for the cynomolgus macaque was reported in 2007 by Kikuchi *et al*.^41^. In their work, they crossed-amplified 148 SSR markers selected from human genome database, and discovered 66 (44%) polymorphic SSR markers in the cynomolgus macaque. Later, Higashino *et al*.^42^ identified an additional 499 polymorphic SSR markers from the BAC library of *M*. *fascicularis*. They analysed the genetic polymorphisms of cynomolgus macaques originated from Indonesia, the Philippines and Malaysia using these SSR markers. In both studies, the SSR markers employed were derived from genomic DNA regions. The polymorphic genic-SSR markers identified in this study is a good addition to complement existing SSR markers to provide more markers for the investigation of the genetic structure of wild macaque populations.

## Materials and Methods

### Ethical clearance

The usage of *M*. *fascicularis* samples in this investigation complied with the animal care regulations and all relevant national laws of Malaysia. Sampling protocols were approved by the Institutional Animal Care and Use Committee (IACUC), University of California, Davis, USA as adopted by the PREDICT Programme in Malaysia, under which the Department of Wildlife and National Park (DWNP) Malaysia is working collaboratively with the EcoHealth Alliance, the Ministry of Health Malaysia, and the Veterinary Services Department, Malaysia.

### *De novo* assembly and functional annotation

A transcriptome dataset was generated from a previous RNA sequencing project of the *M*. *fascicularis* on liver, kidney, lymph node, spleen and thymus^30,31^. We subjected the raw sequencing reads to quality assessment using FASTQC v0.11.2. Illumina co-sequencing positive control (PhiX) sequences were filtered and cleaned sequence reads were subjected to base quality checking (Q ≥ 30). *De novo* sequence assembly was performed using CLC Genomics Workbench version 8.5.1 (CLC Bio-Qiagen, Aarhus, Denmark). We subjected the assembled contigs (average coverage ≥ 10 reads) to annotation by sequence similarity searches with BLAST+ version 2.2.31+^43^ using Blastn against database built with *M*. *fascicularis* (GCF_000364345.1) RNA sequences from the NCBI RefSeq database. Contigs with no match to *M*. *fascicularis* RNA sequences were further searched against database built with *Macaca mulatta* (GCF_000772875.2) protein sequences using Blastp program. Sequences with no match to *M*. *mulatta* protein sequences were then searched against database built with *Homo sapiens* (GRCh38) protein sequences. Contigs with no match were further examined using protein similarity search against SwissProt database.

### Identification and classification of genic-SSRs

Genic-SSR identification and classification were performed on the filtered contigs (average coverage ≥ 10 reads) using MIcroSAtellite identification tool (MISA)^44^. The minimum number of repeats for di-, tri-, tetra-, penta-, and hexanucleotides were set at six, five, five, four, and four, respectively. Categorization of perfect, compound and complex SSRs were as follows. Perfect: consisting of a single repeat of *n* units; compound perfect: consisting of two or more alternate tandem repeats of *n* units each; complex: consisted of repeats that varied in motifs by a single unit/consisted of alternate repeat motifs interspersed within a single region/consisted of two simple perfect motifs separated by nonrepeating sequences of variable length.

### Primer design

Contig sequences containing SSRs identified from the transcriptome dataset were employed for primer development using Primer3 software^45^. We focused on candidate SSR sequences of perfect di- and trinucleotides with repeat numbers ≥10 and with only one SSR presents in each contig for primer design. All contig sequences used for primer design were checked against genomic sequences to predict the location of introns. Three-hundred SSR primer pairs were designed. All the contigs used for SSR primers design were checked for functional annotation where a cut-off value of *E* < 1e^−15^ was used.

### Sampling, DNA extraction, PCR amplification, and electrophoresis

Thirty genic-SSR primer pairs selected randomly from the 300 pairs designed were used for initial screening on the DNA samples of 26 *M*. *fascicularis* individuals. Primers were selected randomly among those that have self- and cross- primer complimentary values of less than 3, low tendency to form secondary structures and 3′- complimentary value of less than 3. To test the robustness of the markers, samples were obtained from nine states in Peninsular Malaysia (Table 2) with the permission and collaboration of DWNP. Three samples from each state were obtained except Terengganu (2 samples). Genomic DNA samples of 24 *M*. *fascicularis* individuals were provided in the form of extracted DNA. Two DNA samples were isolated from liver tissue samples provided by DWNP using QIAamp DNA mini kit (Qiagen, Germany) according to the manufacturer’s protocol.

**Table 2:** GPS locations of the *M*. *fascicularis* samples used.

No.	State	GPS
1	Perlis	247716 734307, 251413 736659, 243353 722458
2	Kedah	209354 699281, 208481 698262, 210009 704813
3	Pulau Pinang	QU263935 WMR615425, QU258571 WMR589429, QU258571 WMR589429
4	Perak	281927 548646, 335311 503907, 285403 468407
5	Selangor	398663 351330, 395891 344432, 389528 356202
6	Negeri Sembilan	485958 311210, 476472 305602, 480356 318551
7	Kelantan	QU435536 WMR605751, QZ437054 WMR552482, QZ438147 WMR539413
8	Pahang	489719 398997
9	Terengganu	586162 555216, 564609 591740

PCR was performed in 10 µl reaction volumes containing 10 ng of genomic DNA, 2.0 µM of each primer, 1$\times$PCR buffer, 2.5 mM MgCl_2_, 0.2 mM dNTPs, and 1 U *Taq* polymerase (Promega, USA), in a thermal cycler T100 (Bio-Rad, USA). Gradient PCR protocol under the following conditions was employed: a single cycle of initial denaturation at 95 °C for 5 minutes, followed by 35 cycles of denaturation at 95 °C for 1 minute, annealing at X °C for 30 seconds, extension at 72 °C for 5 minutes, and ended with a single cycle of final extension at 72 °C for 5 minutes. X denotes the different annealing temperatures (*T*_a_) used for different primers (Table 3). Primers that were not successfully amplified or produced multiple bands were further tested using touchdown PCR with 1 °C decrements starting from 60 °C. PCR products were separated on a 2.0% agarose gel and 8.0% non-denaturing polyacrylamide gel stained with EtBr (0.5 µg/ml). To further confirm the presence of targeted SSRs in the amplified products, PCR products with the expected fragment sizes were sequenced on an ABI 3730 through services provided by First Base Sdn. Bhd. (Seri Kembangan, Malaysia). Sequences obtained were compared with the contig sequences that the primers were designed from and the targeted SSR repeats were also identified.

**Table 3:** The 30 genic-SSR primers used in preliminary screenings on 26 macaque DNA samples.

Primer ID	Primer sequence 5′ → 3′	(Repeat motif)_n_	Product size (bp)	*T*_a_ (°C)
MF013	F:ATCTGTGATGATGGTAAGGA R:GATGGTAACTTGGGTGAGAG	(TGG)_10_	250	52
MF016	F:CCTTAGAGATAGGAAGAAGA R:ATACACACATACACCCTTAC	(GT)_16_	336	48
MF017	F:GGTGAGATTGTAAAGATAGAGG R:AAATGTGCTGGAGAAACC	(TC)_12_	400	54
MF021	F:TGAAGTGGCTGAGGATAG R:AAAGAGGGAACAAACTGG	(TG)_13_	409	53
MF029	F:TCGCTCACTCATTTCTCTGT R:TCACTGTTCAAGGTAGTATGGA	(GT)_13_	340	51
MF069	F:AAACAGGCTTAGATAGGTTC R:TTGGTGATAGATACGATGAG	(CA)_12_	407	51
MF075	F:TGATGATGAGGAAAGGATGAAG R:CCTGGGAAACAAGAGCAAA	(TG)_10_	499	NA
MF080	F:ATTCTGCTTCAGTGTTTGAG R:CTTCATTCCTTTCCTCTATG	(TG)_13_	293	51
MF095	F:GAAAGGGAAATGTAGGAAG R:CTCTCCAAACTCACACCT	(GT)_17_	362	50
MF102	F:CCTCTCCACTCCATCTAC R:GTCAGTTACAGCATTTTGAG	(CA)_13_	479	49
MF113	F:GATACTTGGCATTGGTTGTG R:CACCTCTGTTCTTCTCTGTTG	(CA)_17_	421	57
MF121	F: CTTCATCTGCTCATTCATTC R: CTACATACTTGCCCTTATCAC	(TG)_15_	479	54
MF130	F:GCAGTCAAACCTATTCCTTC R:TCAGAAACCCTCACTCAAAC	(GT)_10_	446	56
MF147	F:TTGACTATTACGGTTTCAGG R:GTTCTTTGATGTGAGGAATG	(AC)_12_	360	53
MF149	F:GTTTGTTCTGTGGCGTGTG R:TAAGCGGTCTCTCTGTTTCC	(GA)_11_	379	NA
MF176	F:AGACCCCGCTTTCCACTAC R:CAGCACAAGACTCCATCTCAA	(AC)_12_	284	60
MF188	F:CTCTGTGGGACCTCTTCTTC R:TCCGTTTGTATGAGTCTGTG	(AC)_11_	457	57
MF197	F:GCAGCAGTGAATAAAAGAAG R:CTGAAACACACGAACTACAC	(AT)_10_	324	52
MF209	F:GCCTGACACTTCCCATCAC R:ATTTCATCCTGTGCTTTGGT	(AC)_16_	318	NA
MF225	F:CTCCCTGTCTCCTTTATCAC R:TTTCTTCCAGTTTCTGTTGG	(AC)_10_	335	54
MF242	F:AAAGAACCATCTACCAAACC R:ATGAAAGCCATTGACACTAC	(TG)_14_	492	54
MF248	F:TTCTTCATTCTGCTCTGTTG R:GCCTATTCTACTCTTGTCATTC	(AC)_13_	294	NA
MF255	F:TTCTGTGGCTTTGGTTTATG R:TGTCAGGGATTGTGAGATTG	(GT)_12_	350	57
MF259	F:AAGCATTCCTCTTAGCAC R:CAAATCGCACAACATCTC	(CA)_10_	319	54
MF261	F:ACCCATTGCTTCCTCTCC R:GGTGTTATTTGTGGTAGTTTGG	(CT)_18_	273	56
MF267	F:CAGTATGATTTCCCATTACC R:AGTTCTGTTTCTCTGTTGTG	(AAC)_11_	353	NA
MF268	F:CTTTGTTCTGCCTTCCTC R:GTGAAACCCCGTAAACTC	(GT)_11_	392	54
MF272	F:GTGATAAGACAGGACAGAGG R:ATAACTACTCCCATTCCAAC	(TG)_10_	376	53
MF273	F:GTTTCTTGCTGATTTCTTCC R:GTCCCACCACTTTGATTTAG	(TG)_11_	441	55
MF279	F:AAGGATAGGAAGATGGTAAG R:GAAAAGGGAAGAGAAAGTG	(TC)_10_	262	51

F, Forward primer; R, Reverse primer; NA, no amplification; *T*_a_, annealing temperature.

### Genetic diversity analysis

The 26 individual macaque samples from the nine states in Peninsular Malaysia were arbitrarily divided into two populations, the East Coast and West Coast populations, taking into consideration the Titiwangsa Range as a potential geographical barrier for gene flow between populations on both coasts. The East Coast population comprised of eight individuals from three states (Kelantan, Terengganu and Pahang), while the West Coast population consisted of 18 individuals from six states (Perlis, Kedah, Pulau Pinang, Perak, Selangor and Negeri Sembilan). SSR banding patterns were analyzed with PopGene version 1.32^46^ and Cervus version 3.0.7^47^ to calculate the number of alleles (*N*_A_), observed heterozygosity (*H*_O_), expected heterozygosity (*H*_E_), fixation index (*F*_ST_), and polymorphic information content (PIC).

## Supplementary information

Supplementary information

## Data Availability

The transcriptome data have been deposited in the NCBI Short Read Archive database under accession SRP096937 and SRX2499144-SRX2499147.
