# Supplementary material for: Discovery of novel genic-SSR markers from transcriptome dataset of an important non-human primate, Macaca fascicularis
Source: Sci Rep. 2019 Jun 11;9:8504. doi: 10.1038/s41598-019-44870-4 (PMC6560038; doi:10.1038/s41598-019-44870-4)
Supplement: Supplementary file 1 — Supplementary information [file 41598_2019_44870_MOESM1_ESM.pdf]

**Discovery of novel genic-SSR markers from transcriptome dataset of an important non-human primate, *Macaca fascicularis***

WENDY CHANG
*Department of Biology, Faculty of Science, Universiti Putra Malaysia, 43400 Serdang, Selangor, Malaysia*
*Email: wendychangwc@gmail.com*

J. EE-ULI
*Department of Cell and Molecular Biology, Faculty of Biotechnology and Biomolecular Sciences, Universiti Putra Malaysia, 43400 Serdang, Selangor, Malaysia*
*Email: joeyeeuli@gmail.com*

W. L. NG
*China-ASEAN Institute of Marine Sciences, Xiamen University Malaysia, Jalan Sunsuria, Bandar Sunsuria, 43900 Sepang, Selangor Darul Ehsan*
*Email: weilun.ng@xmu.edu.my*

JEFFRINE J. ROVIE-RYAN
*National Wildlife Forensic Laboratory (NWFL), Department of Wildlife and National Parks, KM 10, Jalan Cheras, 56100 Kuala Lumpur, Malaysia*
*Email: jeffrine@wildlife.gov.my*

S. G. TAN
*Department of Cell and Molecular Biology, Faculty of Biotechnology and Biomolecular Sciences, Universiti Putra Malaysia, 43400 Serdang, Selangor, Malaysia*
*Email: sgtan_98@yahoo.com*

*CORESPONDING AUTHOR
CHRISTINA S. Y. YONG*
*Department of Biology, Faculty of Science, Universiti Putra Malaysia, 43400 Serdang, Selangor, Malaysia*
*Email: chrisyong@upm.edu.my*
*Telephone: +60389466730*
*Fax: +60386567454*
*ORCID:0000-0002-1399-971X*

Supplementary information

Supplementary Table S1 - Details of 300 novel genic-SSRs markers developed in this study.

Supplementary Table S2 - Comparison of genetic diversity estimates for West Coast and East Coast Peninsular Malaysia populations of *M. fascicularis.*

Supplementary Table S3. Overall genetic diversity estimates at each polymorphic SSR loci.

Table S1. Details of 300 novel genic-SSRs markers developed in this study.

| Primer ID | Primer Sequence 5’→ 3’ | Repeat motif | Product Size (bp) | Tm (°C) | Functional Annotation |
|---|---|---|---|---|---|
| MF001 | F: TATTTTCCTTGGGGTGCT<br>R:ACGAATCTTGGGTATTTAGC | $(CA)_{10}$ | 296 | 55.56<br>53.63 | PREDICTED: *Macaca fascicularis* polypeptide N-acetylgalactosaminyltransferase 1 (GALNT1), mRNA |
| MF002 | F: GTGGAATCTGTGTTCTCATT<br>R:TCAGGTATCGGGTTTATTAC | $(AC)_{11}$ | 329 | 52.30<br>51.87 | PREDICTED: *Macaca fascicularis* LIM domain containing preferred translocation partner in lipoma (LPP), transcript variant X8, mRNA |
| MF003 | F: GTTCTTTGGAATCTGTGG<br>R: ATACACGGGGAATGTCTA | $(GT)_{13}$ | 388 | 50.96<br>50.90 | PREDICTED: *Macaca fascicularis* transmembrane 9 superfamily member 3 (TM9SF3), mRNA |
| MF004 | F:GGACAATAGAGCGAGACTTC<br>R:CTCACACATCCTTCATCCAG | $(AC)_{10}$ | 339 | 54.12<br>56.52 | PREDICTED: *Macaca fascicularis* Kruppel-like factor 7 (ubiquitous) (KLF7), transcript variant X4, mRNA |
| MF005 | F:GGGTGAGGTTGTGAGTAGAAGA<br>R:AGGCTGAGGCACGAGAAT | $(TTG)_{10}$ | 401 | 58.31<br>58.48 | PREDICTED: *Macaca fascicularis* nucleoporin 58kDa (NUP58), transcript variant X4, mRNA |
| MF006 | F:TAAGCACAGGGTTGATAGTG<br>R:AAAGCCAGGACAAACAGAT | $(GT)_{13}$ | 272 | 53.83<br>54.74 | PREDICTED: *Macaca fascicularis* KIAA0226-like ortholog (KIAA0226L), transcript variant X2, mRNA |
| MF007 | F:AGGTGTATCTGTGTATCTGTG<br>R:AGTGATTGTAGTAGGTGTCTG | $(TG)_{11}$ | 359 | 49.83<br>49.19 | PREDICTED: *Macaca fascicularis* glucosamine (N-acetyl)-6-sulfatase (GNS), transcript variant X1, mRNA |
| MF008 | F:TTTCTTCCCTCCCTCTATG<br>R:AACATCTGCCTGCTATCTG | $(AC)_{11}$ | 359 | 54.31<br>53.82 | PREDICTED: *Macaca fascicularis* ring finger protein 38 (RNF38), transcript variant X18, mRNA |
| MF009 | F:ATCACTGTTTCATAGCATCC<br>R:AAGAACACATTCACCACTTG | $(GT)_{10}$ | 338 | 52.52<br>52.23 | PREDICTED: *Macaca fascicularis* actin binding LIM protein 1 (ABLIM1), transcript variant X26, mRNA |
| MF010 | F:CAGACCTATCTCAGGAACAC<br>R:AAGAACACATTCACCACTTG | $(AC)_{10}$ | 426 | 52.03<br>53.45 | PREDICTED: *Macaca fascicularis* zinc finger, RAN-binding domain containing 1 (ZRANB1), transcript variant X1, mRNA |
| MF011 | F:ACTGATACTGATGGACACTG<br>R:CTATTTCCTATTGCTGGAGT | $(CA)_{15}$ | 369 | 50.45<br>51.30 | PREDICTED: *Macaca fascicularis* protein tyrosine phosphatase type IVA, member 2 (PTP4A2), transcript variant X4, mRNA |
| MF012 | F:TCCTGTTGTTGATGTGATAC<br>R:ATTAGTGGATAGGTTCTTCC | $(TA)_{10}$ | 483 | 51.47<br>49.99 | PREDICTED: *Macaca fascicularis* PNN-interacting serine/arginine-rich protein (PNISR), transcript variant X1, mRNA |
| MF013 | F:ATCTGTGATGATGGTAAGGA<br>R:GATGGTAACTTGGGTGAGAG | $(TGG)_{10}$ | 250 | 52.73<br>54.59 | PREDICTED: *Macaca fascicularis* cell adhesion molecule 1 (CADM1), transcript variant X4, mRNA |
| MF014 | F:GGGCAATCAAACACCTTT<br>R:CTGGACAAAACCCCACTAC | $(TG)_{12}$ | 331 | 55.91<br>55.40 | PREDICTED: *Macaca fascicularis* activating transcription factor 7 interacting protein (ATF7IP), |

| | | | | | |
|---|---|---|---|---|---|
| | | | | | transcript variant X10, mRNA |
| MF015 | F:GATTGTTGTTTACTCCCAGA<br>R:CCTTGGTTCTACTCTTACCC | $(GT)_{15}$ | 428 | 52.62<br>53.05 | PREDICTED: *Macaca fascicularis* v-ets avian erythroblastosis virus E26 oncogene homolog 1 (ETS1), transcript variant X2, mRNA |
| MF016 | F:CCTTAGAGATAGGAAGAAG<br>A<br>R:ATACACACATACACCCTTAC | $(GT)_{16}$ | 336 | 48.02<br>46.98 | PREDICTED: *Macaca fascicularis* A-kinase anchor protein 2 (LOC102133073), transcript variant X6, mRNA |
| MF017 | F:GGTGAGATTGTAAAGATAG<br>AGG<br>R:AAATGTGCTGGAGAAACC | $(TC)_{12}$ | 400 | 52.43<br>53.37 | PREDICTED: *Macaca fascicularis* RAP2C, member of RAS oncogene family (RAP2C), transcript variant X2, mRNA |
| MF018 | F:ACCTGTAGTCCCAAAGTG<br>R:TAAATACCTCTTCTGTAGGC | $(AC)_{12}$ | 286 | 50.43<br>48.98 | PREDICTED: *Macaca fascicularis* integrator complex subunit 10 (INTS10), transcript variant X5, mRNA |
| MF019 | F:GTGTCCATCCACTGTAAAC<br>R:TCTTCTCACTTTGTCATCC | $(TG)_{10}$ | 376 | 50.72<br>50.61 | PREDICTED: *Macaca fascicularis* potassium channel, inwardly rectifying subfamily J, member 10 (KCNJ10), mRNA |
| MF020 | F:AAAGAATGAGGAGGACGA<br>R:GTCTGGGTGGAACTTGAG | $(AGC)_{12}$ | 454 | 52.95<br>53.65 | PREDICTED: *Macaca fascicularis* nucleobindin 1 (NUCB1), mRNA |
| MF021 | F:TGAAGTGGCTGAGGATAG<br>R:AAAGAGGGAACAAACTGG | $(TG)_{13}$ | 409 | 51.82<br>52.49 | PREDICTED: *Macaca fascicularis* A-kinase anchoring protein 11 (AKAP11), transcript variant X3, mRNA |
| MF022 | F:GTTGCCGTGTGCTTACTG<br>R:ATTTCTTGGCTCTGCTATTG | $(GT)_{12}$ | 489 | 56.25<br>54.81 | PREDICTED: *Macaca fascicularis* ATPase, aminophospholipid transporter, class I, type 8B, member 1 (ATP8B1), transcript variant X4, mRNA |
| MF023 | F:ATTTGTGCTGCCTGTTTG<br>R:GCTTTCTAAGTTTGCCAGTC | $(AC)_{11}$ | 286 | 55.65<br>54.42 | PREDICTED: *Macaca fascicularis* butyrophilin like 9 (BTNL9), mRNA |
| MF024 | F:GTCTACAATCTTCTGCTCTG<br>R:CCTCCATCTACCTACACAC | $(GT)_{13}$ | 405 | 49.88<br>49.97 | PREDICTED: *Macaca fascicularis* schlafen family member 5 (SLFN5), transcript variant X1, mRNA |
| MF025 | F:GTTGGTTGAGGTGTGTCTC<br>R:TTTCAGGACTTTGGAGTTC | $(AC)_{13}$ | 463 | 53.55<br>52.64 | PREDICTED: *Macaca fascicularis* dedicator of cytokinesis 1 (DOCK1), transcript variant X4, mRNA |
| MF026 | F:CTCTGTGTGTTTAGGGAGT<br>R:TAAGGGCTGGTAGTAGAAG | $(AC)_{11}$ | 304 | 49.76<br>49.73 | PREDICTED: *Macaca fascicularis* GC-rich promoter binding protein 1 like 1 (GPBP1L1), transcript variant X3, mRNA |
| MF027 | F:GATTTAGTGTAGGTAGTGTT<br>GG<br>R:AGAAGATGTGATGCTGAGA<br>C | $(CT)_{10}$ | 361 | 50.90<br>52.57 | PREDICTED: *Macaca fascicularis* RNA binding motif, single stranded interacting protein 1 (RBMS1), transcript variant X9, mRNA |
| MF028 | F:ATGTTTGTGTGGTGTTGGTA<br>GT<br>R:GAACTCATCCCATTCTGACC | $(GT)_{10}$ | 448 | 56.89<br>56.89 | PREDICTED: *Macaca fascicularis* arf-GAP with GTPase, ANK repeat and PH domain-containing protein |

| | | | | | |
|---|---|---|---|---|---|
| | | | | | 5-like (LOC102116722), transcript variant X1, mRNA |
| MF029 | F:TCGCTCACTCATTTCTCTGT<br>R:TCACTGTTCAAGGTAGTATGGA | $(GT)_{13}$ | 340 | 56.07<br>54.91 | PREDICTED: *Macaca fascicularis* BRCA1 associated protein (BRAP), transcript variant X3, mRNA |
| MF030 | F:TAAAGCAAGAGCAACTGATG<br>R:AGAAAGAGACAGAGGAGAGG | $(GT)_{10}$ | 324 | 54.31<br>52.70 | PREDICTED: *Macaca fascicularis* cyclin-dependent kinase 14 (CDK14), transcript variant X1, mRNA |
| MF031 | F:TTTCTCCTGGCTGTCTCTG<br>R:TTCCCTTTGGCAGTAAATAG | $(AC)_{12}$ | 317 | 56.54<br>54.69 | PREDICTED: *Macaca fascicularis* B-cell CLL/lymphoma 2 (BCL2), mRNA |
| MF032 | F:TGTGAGGACAGACAGAGAAG<br>R:AAATGAAAGGGAGAAAGGTC | $(AC)_{17}$ | 400 | 53.65<br>54.93 | PREDICTED: *Macaca fascicularis* uncharacterized LOC107129844 (LOC107129844), transcript variant X1, mRNA |
| MF033 | F:TATGCTTGTGTGTAGTTGC<br>R:AGGGTGAGGTTTTCTTATC | $(TG)_{11}$ | 360 | 50.34<br>50.70 | No hit |
| MF034 | F:CCTTATGTTCCTGAGTTATG<br>R:CTGGGTGACAGAGGTAGA | $(TG)_{10}$ | 332 | 49.83<br>51.38 | PREDICTED: *Macaca fascicularis* G protein-coupled receptor associated sorting protein 1 (GPRASP1), transcript variant X5, mRNA |
| MF035 | F:TCCTACTGGATGAAGAGAAG<br>R:GACAAATGAGATGGAAACTC | $(TG)_{13}$ | 454 | 51.92<br>51.96 | PREDICTED: *Macaca fascicularis* 2'-5'-oligoadenylate synthetase 3 (OAS3), transcript variant X1, mRNA |
| MF036 | F:GTATGTGAAAGGCAGTGG<br>R:ACAATCCGAACCCAGTAT | $(GT)_{11}$ | 479 | 52.07<br>52.54 | PREDICTED: *Macaca fascicularis* AF4/FMR2 family member 1 (AFF1), transcript variant X6, mRNA |
| MF037 | F:AGTTCCACTTTGGTTATTGG<br>R:TTAGAAGAGATTTGCCTTGG | $(AC)_{18}$ | 288 | 54.24<br>54.78 | *Macaca fascicularis* steroid sulfatase (microsomal), isozyme S (STS), mRNA |
| MF038 | F:ATTATTCCAGTGAGCCAGTT<br>R:AAACCACCCTTGTATTTGG | $(TG)_{15}$ | 452 | 53.87<br>54.91 | PREDICTED: *Macaca fascicularis* membrane associated ring-CH-type finger 7 (MARCH7), transcript variant X4, mRNA |
| MF039 | F:ACTTTCATCCTCAGTTTCC<br>R:AGTAATCCTCACTCCAGGTA | $(GT)_{10}$ | 498 | 51.47<br>51.23 | PREDICTED: *Macaca fascicularis* DDB1 and CUL4 associated factor 12 (DCAF12), transcript variant X3, mRNA |
| MF040 | F:TTCCTCTACCAGAACTAAGC<br>R:CAAGATACTACCAGTCAACATC | $(TG)_{11}$ | 463 | 51.41<br>51.50 | PREDICTED: *Macaca fascicularis* sulfatase 2 (SULF2), transcript variant X3, mRNA |
| MF041 | F:TGTTTGTTTACCTGTGCTC<br>R:CACTCCCTGACCTATGAG | $(AG)_{11}$ | 261 | 51.99<br>50.88 | PREDICTED: *Macaca fascicularis* myelin protein zero (MPZ), mRNA |
| MF042 | F:ACACAGTAAGCCAGAACAC<br>R:CTCCTGACACACCCTCTA | $(AC)_{13}$ | 432 | 50.75<br>51.38 | PREDICTED: *Macaca fascicularis* zinc finger protein 655 (ZNF655), transcript variant X9, mRNA |
| MF043 | F:GCCTATGACAAGTATGGAG<br>R:ACAAGTTCTCTGGAAGTGA | $(AC)_{12}$ | 361 | 50.43<br>50.23 | PREDICTED: *Macaca fascicularis* peroxisomal biogenesis factor 19 (PEX19), transcript variant X1, mRNA |
| MF044 | F:GACCTTCCTCAGTCATTTCT | $(CA)_{14}$ | 379 | 53.27 | PREDICTED: *Macaca fascicularis* |

| | | | | | |
|---|---|---|---|---|---|
| | R:AATCCTAAACACAACCAGAC | | | 51.60 | colony stimulating factor 2 receptor beta common subunit (CSF2RB), transcript variant X1, mRNA |
| MF045 | F:ACTCAAAGCCATCCAACCTC<br>R:CCAGAATCACATCCAGAAGG | $(GT)_{16}$ | 399 | 59.14<br>58.07 | PREDICTED: *Macaca fascicularis* transducer of ERBB2, 2 (TOB2), mRNA |
| MF046 | F:ACTTTGTGTCAGTTGGTTTC<br>R:CTCTTTCTTGATTCTTGGTG | $(AT)_{10}$ | 324 | 52.53<br>52.50 | PREDICTED: *Macaca fascicularis* glypican 6 (GPC6), transcript variant X6, mRNA |
| MF047 | F:GGAAGAATCCAAGTGTGAG<br>R:ACCTAACCTGACCTCTGAC | $(GT)_{11}$ | 496 | 52.88<br>51.30 | No hit |
| MF048 | F:GAAGAGGAACAGAAGCAG<br>R:CTTGTAATGAGGGTGACAT | $(TG)_{13}$ | 449 | 50.50<br>50.48 | No hit |
| MF049 | F:GGAGAACAAAGAGACAGTG<br>R:ATACTACAGCGAGGAGGTG | $(AG)_{12}$ | 395 | 50.23<br>52.78 | PREDICTED: *Macaca fascicularis* platelet derived growth factor subunit A (PDGFA), transcript variant X2, mRNA |
| MF050 | F:TTGGCATTCTTCGCTGATT<br>R:GGCTGCTCACACAACAGAC | $(AC)_{12}$ | 449 | 59.37<br>57.87 | PREDICTED: *Macaca fascicularis* calcium/calmodulin-dependent protein kinase II inhibitor 1 (CAMK2N1), mRNA |
| MF051 | F:TTACAGAGCAGTTAGGAATG<br>R:CAGTTCAGGTATCCAGGTA | $(TG)_{17}$ | 301 | 50.63<br>50.72 | PREDICTED: *Macaca fascicularis* ligand-dependent corepressor (LOC102145999), transcript variant X5, mRNA |
| MF052 | F:GTGTGAAGTCCCTCCTGATA<br>R:GATTTTTCTCCTGCTCCATC | $(GT)_{13}$ | 392 | 55.01<br>56.37 | PREDICTED: *Macaca fascicularis* S100P binding protein (S100PBP), transcript variant X4, mRNA |
| MF053 | F:GTGTTACCTCCTCTGAATGG<br>R:GCCACAAAGATAGCCACAG | $(AC)_{14}$ | 310 | 54.59<br>56.83 | No hit |
| MF054 | F:ATTCCTCTTCTCACCACTTC<br>R:TGTAACTGACTTTTGTGTGC | $(AC)_{11}$ | 250 | 53.27<br>52.12 | LINE-1 retrotransposable element ORF2 protein OS=Homo sapiens PE=1 SV=1 |
| MF055 | F:ATAGAGACAAAGGGAGAAAG<br>R:TAACAAGTCCACAACCTG | $(GT)_{13}$ | 432 | 50.30<br>49.68 | No hit |
| MF056 | F:CGACATTATCTTGCTCCTAC<br>R:TCCAAAGGTGTTTACTGAAG | $(CA)_{22}$ | 446 | 52.56<br>52.93 | No hit |
| MF057 | F:GCAGTGAGCCGAGATTGTA<br>R:AACTTCCTCTGGTGATTTAGC | $(AC)_{12}$ | 278 | 57.49<br>55.22 | PREDICTED: *Macaca fascicularis* phosphoinositide-3-kinase regulatory subunit 5 (PIK3R5), transcript variant X2, mRNA |
| MF058 | F:GTGAAGACAGATGAAAGC<br>R:TACACTGGGAAGTTTGTTAC | $(GT)_{14}$ | 355 | 48.18<br>50.16 | No hit |
| MF059 | F:GTGGTAAGGTCGTTGTCG<br>R:ACTACTCCGTGCCCATCT | $(AC)_{10}$ | 470 | 54.82<br>55.50 | PREDICTED: *Macaca fascicularis* homeobox protein NANOG-like (LOC102143010), mRNA |
| MF060 | F:TATTTTCCTCTGCTATCTGC<br>R:TGATGTGACTGTAAACCTTG | $(AT)_{12}$ | 448 | 52.41<br>51.83 | PREDICTED: *Macaca fascicularis* taxilin gamma (TXLNG), transcript variant X3, mRNA |
| MF061 | F:AACGCACACACATCATACAC<br>R:AGGATTAGAACCACATCAGTG | $(CA)_{12}$ | 484 | 55.25<br>54.20 | PREDICTED: *Macaca fascicularis* RAR-related orphan receptor A (RORA), transcript variant X2, mRNA |

| | | | | | |
|---|---|---|---|---|---|
| MF062 | F:TGTCCTCTCAACATAACTGT<br>R:GGTGATACTGGCTTCATAG | $(AAC)_{10}$ | 422 | 50.28<br>50.43 | PREDICTED: *Macaca fascicularis* suppressyn (LOC107129059), mRNA |
| MF063 | F:AAAGTCCACCAGTTAGAGC<br>R:AGAGACACACTCAAGGAGAC | $(TG)_{11}$ | 343 | 51.83<br>51.44 | No hit |
| MF064 | F:GGAACATCCCTCCTTCTTTG<br>R:ATTATCCCACGCAGGTCTAC | $(GT)_{12}$ | 478 | 58.57<br>56.63 | No hit |
| MF065 | F:CAAAGATGAGACCTATGACC<br>R:GTATGCCTTCAAACTGCTTA | $(GT)_{10}$ | 486 | 52.03<br>53.27 | No hit |
| MF066 | F:TGAATGTCTCTTGCTTCC<br>R:CCTTATTTTCTGGCACTAC | $(AT)_{13}$ | 457 | 51.74<br>49.97 | PREDICTED: *Macaca fascicularis* flavin containing monooxygenase 5 (FMO5), mRNA |
| MF067 | F:AAACAGGCTTAGATAGGTTC<br>R:TTGGTGATAGATACGATGAG | $(CA)_{12}$ | 407 | 50.48<br>50.96 | PREDICTED: *Macaca fascicularis* family with sequence similarity 227 member B (FAM227B), transcript variant X1, mRNA |
| MF068 | F:CGACTCTTCTGTCTTGCT<br>R:CTTCGTGACTTCCCTATG | $(GT)_{13}$ | 308 | 51.04<br>51.25 | PREDICTED: *Macaca fascicularis* basic helix-loop-helix family member e40 (BHLHE40), mRNA |
| MF069 | F:GCTACAGAGGATACGAGGAG<br>R:CACAGAGCAAACAGTCATTG | $(AC)_{12}$ | 437 | 54.17<br>55.28 | No hit |
| MF070 | F:TAAGGCTGTTAGGTTGTGTG<br>R:CTTGGTCTGGCTGGATTC | $(GT)_{15}$ | 254 | 53.93<br>56.58 | PREDICTED: *Macaca fascicularis* MLX interacting protein (MLXIP), mRNA |
| MF071 | F:AGCAGGATTCAGATAGGATA<br>R:AAGGAAAGAAGAGCCAAC | $(AG)_{10}$ | 329 | 51.50<br>51.85 | No hit |
| MF072 | F:CAAACCTTGAGACGATTGG<br>R:AGGAGGGACAGTTAGTAGGAG | $(AC)_{11}$ | 324 | 56.64<br>54.25 | PREDICTED: *Macaca fascicularis* vestigial like family member 3 (VGLL3), transcript variant X4, mRNA |
| MF073 | F:TGATGATGAGGAAAGGATGAAG<br>R:CCTGGGAAACAAGAGCAAA | $(TG)_{10}$ | 499 | 59.13<br>58.83 | PREDICTED: *Macaca fascicularis* uncharacterized LOC102119301 (LOC102119301), transcript variant X1, ncRNA |
| MF074 | F:TGAAGTTGTGTGGTGTAGTG<br>R:GGATTTGAGGTTAGGTATTC | $(GT)_{10}$ | 297 | 52.68<br>50.45 | No hit |
| MF075 | F:TCTGTATCTCTTCTCACTTG<br>R:GTGTGTGTCTGTGTTCGT | $(AC)_{13}$ | 485 | 47.38<br>49.97 | No hit |
| MF076 | F:ACAAGATACAAAGCGAGAC<br>R:TCATAAGTTGGGAAGTGC | $(AC)_{15}$ | 386 | 50.19<br>51.73 | PREDICTED: *Macaca fascicularis* thromboxane A2 receptor (TBXA2R), mRNA |
| MF077 | F:TTTAGCATCTCTGGTCTTAG<br>R:AAGTAGGGTCCAAACAAC | $(TG)_{18}$ | 293 | 49.88<br>49.57 | PREDICTED: *Macaca fascicularis* forkhead box J3 (FOXJ3), transcript variant X7, mRNA |
| MF078 | F:ATTCTGCTTCAGTGTTTGAG<br>R:CTTCATTCCTTTCCTCTATG | $(TG)_{13}$ | 293 | 53.00<br>51.59 | No hit |
| MF079 | F:CAGGCACAAAGTATTCCTAC<br>R:AAATCTGGCACTAATCACAC | $(TG)_{10}$ | 264 | 52.49<br>52.64 | No hit |
| MF080 | F:CTTTACTCTTTCAGGTCACT | $(TC)_{11}$ | 390 | 50.86 | PREDICTED: *Macaca fascicularis* |

| | | | | | |
|---|---|---|---|---|---|
| | C<br>R:ACATTCACATACACAAGCAC | | | 51.62 | protocadherin alpha-C2 (LOC102136630), transcript variant X16, mRNA |
| MF081 | F:CCATCACCTCACATAGTTAC<br>R:ATTGAACCCAGACCTACA | (TG)$_{21}$ | 332 | 50.18<br>50.81 | No hit |
| MF082 | F:AGATGGCTCTTGTGTGAAAG<br>R:CTTACCTCATTCTTCTTCTGC | (CA)$_{10}$ | 413 | 55.45<br>53.53 | No hit |
| MF083 | F:TTGACCTTTGGGGAGTTTG<br>R:AAGAATGAAGCACACCTACAAG | (TC)$_{15}$ | 271 | 58.53<br>56.22 | PREDICTED: *Macaca fascicularis* sorting nexin 13 (SNX13), transcript variant X6, mRNA |
| MF084 | F:AATAAGTCCATTACCACAGG<br>R:TAGATGTTGCTCCTCTTCTG | (TG)$_{13}$ | 491 | 51.68<br>53.09 | PREDICTED: *Macaca fascicularis* collagen type VIII alpha 1 (COL8A1), transcript variant X3, mRNA |
| MF085 | F:TGTTAGGAGTGTTGTGTTTC<br>R:GTATTCCATTGTTTGGCTAC | (CT)$_{14}$ | 363 | 50.92<br>52.28 | No hit |
| MF086 | F:ATTTCAGTTTCTCCCAACC<br>R:GGACTCTTTGCTTGTCAGG | (CA)$_{15}$ | 351 | 54.03<br>55.88 | No hit |
| MF087 | F:CAGACATACCCACAGATAAG<br>R:GTGACTTTAGACTGGCTCT | (CA)$_{15}$ | 462 | 50.41<br>49.12 | No hit |
| MF088 | F:AACACACAATCCCAGTCCTC<br>R:ATTCTCAGGCATCAACCGTA | (TG)$_{12}$ | 357 | 57.38<br>58.17 | PREDICTED: *Macaca fascicularis* ATP synthase mitochondrial F1 complex assembly factor 1 (ATPAF1), mRNA |
| MF089 | F:TGGACACACAGACACACAG<br>R:ACAAGCGAGATGAAGAAAC | (AC)$_{10}$ | 363 | 54.39<br>52.85 | PREDICTED: *Macaca fascicularis* PMS1 homolog 2, mismatch repair system component (PMS2), mRNA |
| MF090 | F:TCAGGAGTTTGAGACCAG<br>R:TTGTTTCTGTGTGTGTGG | (AC)$_{13}$ | 269 | 51.54<br>51.43 | PREDICTED: *Macaca fascicularis* uncharacterized LOC102122431 (LOC102122431), ncRNA |
| MF091 | F:GTCCCATCTATTTATCTCTG<br>R:CACTGACCAAACTCAAAC | (TG)$_{11}$ | 435 | 48.70<br>49.03 | No hit |
| MF092 | F:CTGGAAAGATAACGGAAAC<br>R:ACCCACGCAACATTTAGT | (AC)$_{15}$ | 483 | 52.32<br>53.85 | PREDICTED: *Macaca fascicularis* nuclear receptor subfamily 4 group A member 2 (NR4A2), transcript variant X5, misc_RNA |
| MF093 | F:GAAAGGGAAATGTAGGAAG<br>R:CTCTCCAAACTCACACCT | (GT)$_{17}$ | 362 | 51.38<br>50.40 | No hit |
| MF094 | F:GTGGATGCTATTGTAGGAG<br>R:GATGGAGATGGATAATGTG | (AC)$_{10}$ | 344 | 50.43<br>50.72 | No hit |
| MF095 | F:CATTTCTCTTGGAGTGGAAC<br>R:GTCTGCTCACCTTCTCTGAC | (AC)$_{16}$ | 389 | 54.73<br>54.93 | PREDICTED: *Macaca fascicularis* paired box 5 (PAX5), transcript variant X4, mRNA |
| MF096 | F:TTCTCTTGACTGTTGCTCTC<br>R:TAGTTCTTGCCATCTACCAC | (GT)$_{10}$ | 350 | 53.05<br>52.84 | No hit |
| MF097 | F:CTCTCAACCTCTCCTCTCTC<br>R:CACACAAGTTCTCTCAACAG | (GT)$_{12}$ | 254 | 53.07<br>51.47 | PREDICTED: *Macaca fascicularis* CD3d molecule (CD3D), transcript variant X2, mRNA |
| MF098 | F:CCTGCTCTCCTAAAATGAAG<br>R:GCCACTAAAATACCACTCTG | (TG)$_{13}$ | 426 | 54.36<br>52.49 | No hit |
| MF099 | F:GTGTCAGGAAAGGACTCTTG | (CA)$_{10}$ | 260 | 54.79<br>55.38 | PREDICTED: *Macaca fascicularis* mediator complex subunit 13-like |

| | | | | | |
|---|---|---|---|---|---|
| | R:GAAAGCAGATAAAAGCGATG | | | | (MED13L), transcript variant X4, mRNA |
| MF100 | F:CCTCTCCACTCCATCTAC<br>R:GTCAGTTACAGCATTTTGAG | $(CA)_{13}$ | 479 | 49.76<br>50.85 | No hit |
| MF101 | F:TGGGTATGTGTGTGTATCTG<br>R:AAGAAAGTAGGGACGATGAC | $(TG)_{14}$ | 376 | 52.40<br>53.39 | PREDICTED: *Macaca fascicularis* peroxisome proliferator-activated receptor gamma, coactivator 1 beta (PPARGC1B), transcript variant X4, mRNA |
| MF102 | F:CTTTCTGTGTTTTGAGGTG<br>R:CCAGGGTATGTTATTTATCC | $(CA)_{14}$ | 330 | 51.47<br>51.27 | PREDICTED: *Macaca fascicularis* ATM interactor (ATMIN), transcript variant X4, mRNA |
| MF103 | F:CATCAACCAACCAACAGGTG<br>R:TATCCCTTACTCCCTCCCAAC | $(AC)_{11}$ | 349 | 59.85<br>59.29 | PREDICTED: *Macaca fascicularis* family with sequence similarity 104 member B (FAM104B), transcript variant X1, mRNA |
| MF104 | F:CACCCCAGGAGAGAGATTG<br>R:GTTTTGCCGCTTGTGATAC | $(AC)_{10}$ | 421 | 58.14<br>56.79 | PREDICTED: *Macaca fascicularis* mediator complex subunit 25 (MED25), transcript variant X6, mRNA |
| MF105 | F:AATAATGGTGGTAGAAGGTC<br>R:AAATCAAGAAGTGTGAGGTC | $(AC)_{10}$ | 435 | 50.73<br>51.61 | No hit |
| MF106 | F:CAACATACAATACTCCCTTCG<br>R:CTTGACTGCTCTCTTTCCTG | $(TG)_{12}$ | 455 | 54.88<br>55.33 | No hit |
| MF107 | F:CTCACTTTCCATCTTCTGAT<br>R:ATTGCCACTAATAACCTCTC | $(TG)_{13}$ | 394 | 51.70<br>51.09 | No hit |
| MF108 | F:GGTCTGTTTGCCTAATGTTG<br>R:AGTCTTGCTCTGTTGTCCAG | $(TG)_{12}$ | 438 | 55.75<br>55.53 | PREDICTED: *Macaca fascicularis* kelch like family member 42 (KLHL42), mRNA |
| MF109 | F:CCTCCACCTCCTAAAAGTG<br>R:GAGAAAATCACAGCATTCAG | $(GA)_{11}$ | 321 | 54.77<br>53.35 | PREDICTED: *Macaca fascicularis* family with sequence similarity 118 member A (FAM118A), transcript variant X14, mRNA |
| MF110 | F:CTTTAGCCTGTCCAATGTC<br>R:GTCAGTCACAATCAAATCAG | $(TG)_{11}$ | 366 | 53.20<br>51.12 | No hit |
| MF111 | F:TGGTAGAAACTGGTGACTC<br>R:GTCTGTTGGACACTTGAAT | $(TG)_{15}$ | 442 | 50.60<br>50.35 | No hit |
| MF112 | F:CTGTATTGTTTCAAGCCACT<br>R:TCATTTCTGTCATCTCTCAG | $(TG)_{11}$ | 463 | 52.95<br>50.86 | No hit |
| MF113 | F:GATACTTGGCATTGGTTGTG<br>R:CACCTCTGTTCTTCTCTGTTG | $(CA)_{17}$ | 421 | 56.48<br>55.08 | No hit |
| MF114 | F:GAAGCATTTAGAAGGACAC<br>R:ACTCCATAGCAAAGCATAC | $(GT)_{10}$ | 348 | 49.69<br>50.29 | No hit |
| MF115 | F:TAACTTTCCCATCTGTTTCC<br>R:ACTCTTACCTCCTTCCAATC | $(GT)_{12}$ | 447 | 54.28<br>52.45 | No hit |
| MF116 | F:CTCTTTCACTTTTGGGAGTC<br>R:GTTCTTCTGGCTGTCTTATG | $(AC)_{10}$ | 285 | 54.02<br>52.52 | No hit |
| MF117 | F:GGATACTGGGAAAATAACG<br>R:GGGCTAATGTGAATACCTG | $(CA)_{13}$ | 404 | 52.19<br>53.04 | PREDICTED: *Macaca fascicularis* uncharacterized LOC102135239 (LOC102135239), ncRNA |

| | | | | | |
|---|---|---|---|---|---|
| MF118 | F:CTGGTGAAGAACATAAAAGC<br>R:CACAATGAGGAGGATTAGA | $(AC)_{10}$ | 364 | 52.62<br>50.74 | No hit |
| MF119 | F:CCAAAATCTCCCATAAACAC<br>R:AAAAGCAGTAGCAAAAGCAG | $(AC)_{16}$ | 264 | 54.57<br>55.29 | No hit |
| MF120 | F:ATGGTCTTCTACAGTTGG<br>R:TATGAGAGTGTGGTATTGC | $(AC)_{16}$ | 442 | 47.57<br>48.94 | No hit |
| MF121 | F:CTTCATCTGCTCATTCATTC<br>R:CTACATACTTGCCCTTATCAC | $(TG)_{15}$ | 479 | 53.25<br>51.73 | No hit |
| MF122 | F:AGGCTCAAATAACTCCCACT<br>R:TGTGAAGGAAAGTCTGATGG | $(AC)_{13}$ | 306 | 55.48<br>56.18 | No hit |
| MF123 | F:TGCTTTCCCATCATCTCTAC<br>R:AGTTCTCAGTTTGGCACATC | $(AC)_{10}$ | 318 | 55.29<br>55.26 | No hit |
| MF124 | F:ACTCCTGATTCCTAACCTA<br>R:TTTCTCATAGTGCTCCTTC | $(CA)_{12}$ | 345 | 48.61<br>50.23 | No hit |
| MF125 | F:GTCTGCCAAGAAATCAGTTG<br>R:TGAAGAACCACAGTCACAAG | $(TC)_{12}$ | 272 | 55.89<br>54.58 | No hit |
| MF126 | F:GAGAAGAGGAAGAGGATTC<br>R:GGTATGGATGAGGAAATAG | $(TC)_{10}$ | 480 | 50.20<br>49.31 | No hit |
| MF127 | F:TCTTCAGGTATTTCACTTCC<br>R:CAGGTATGTGTATCCTCCTC | $(TC)_{10}$ | 422 | 51.83<br>51.89 | No hit |
| MF128 | F:CCTTTTATCTGAGGTTCTAC<br>R:TTTTCTTCACTGGCTACTC | $(TG)_{12}$ | 478 | 48.26<br>50.37 | No hit |
| MF129 | F:TCTCACTTCACTTCACCTTG<br>R:ATCTGCTCTCCTTTGCTTC | $(CT)_{16}$ | 428 | 53.72<br>54.69 | PREDICTED: *Macaca fascicularis* homeobox C6 (HOXC6), transcript variant X1, mRNA |
| MF130 | F:GCAGTCAAACCTATTCCTTC<br>R:TCAGAAACCCTCACTCAAAC | $(GT)_{10}$ | 446 | 54.00<br>55.24 | PREDICTED: *Macaca fascicularis* signal transducer and activator of transcription 5A (LOC102120556), mRNA |
| MF131 | F:CAAGTAGGTGAAGAGATGG<br>R:AACTGATTGGATGAGACC | $(TG)_{13}$ | 298 | 50.37<br>50.04 | PREDICTED: *Macaca fascicularis* uncharacterized LOC107129272 (LOC107129272), ncRNA |
| MF132 | F:AGTTGCCCATAGTCTGAG<br>R:GCCAGTCATTTATCCTTAG | $(GT)_{15}$ | 328 | 50.71<br>49.84 | No hit |
| MF133 | F:CTATGGTGAGAGATGGTGTG<br>R:GAAGATTTACCCTTTGGAAG | $(TC)_{10}$ | 367 | 53.81<br>53.16 | No hit |
| MF134 | F:TAGGGGAAAATCTGTTTGC<br>R:AGCAGGAAGGAGAATGAAC | $(CT)_{10}$ | 445 | 55.29<br>54.33 | PREDICTED: *Macaca fascicularis* zinc finger protein interacting with K protein 1 (ZIK1), transcript variant X7, mRNA |
| MF135 | F:TTGGACACATCCTTCTTG<br>R:GAATCAAAGCCCTACACC | $(TG)_{14}$ | 481 | 52.44<br>52.87 | No hit |
| MF136 | F:GCAATAAGCACAATAGCAAC<br>R:CGTTAGAAACCACTTGACTG | $(AC)_{10}$ | 384 | 53.73<br>53.44 | No hit |
| MF137 | F:TCACAGCACATACATCTTAC<br>R:CATTACCCAAGGAATCTC | $(AC)_{10}$ | 383 | 49.11<br>50.03 | No hit |

| | | | | | |
|---|---|---|---|---|---|
| MF138 | F:GAGATGGACTTTATTAGCAG<br>R:CAGCAGACAGAGTGTGAC | $(GT)_{14}$ | 379 | 49.24<br>49.90 | PREDICTED: *Macaca fascicularis* uncharacterized LOC102116437 (LOC102116437), ncRNA |
| MF139 | F:GATGGCTTGTTTACTGAGC<br>R:TATGCTTTGTGGTGGACTA | $(AC)_{14}$ | 472 | 53.36<br>52.43 | PREDICTED: *Macaca fascicularis* 25-hydroxycholesterol 7-alpha-hydroxylase (LOC102132453), transcript variant X1, mRNA |
| MF140 | F:GTTTCACACAGACATTGG<br>R:CTTACATTTCCAGCCTTC | $(AC)_{16}$ | 343 | 49.74<br>50.57 | PREDICTED: *Macaca fascicularis* storkhead box 2 (STOX2), transcript variant X5, mRNA |
| MF141 | F:AAATAGGTCAGTCGGTCTC<br>R:TTTAGTTGGTGTTGTGCTG | $(TA)_{10}$ | 364 | 51.52<br>53.04 | PREDICTED: *Macaca fascicularis* uncharacterized LOC102131235 (LOC102131235), transcript variant X2, ncRNA |
| MF142 | F:GATTTGTCCTGAGAGGCTTG<br>R:ATTGTTCCATCTTCGTCCAG | $(TG)_{14}$ | 365 | 57.45<br>57.58 | No hit |
| MF143 | F:ACTATGGAGTTTTGGGTAAG<br>R:TTCTGCTACTGTTTTCTCAC | $(AC)_{15}$ | 364 | 51.06<br>50.51 | No hit |
| MF144 | F:CTTGCTCTTTCTTCCTCTC<br>R:ATAACTTCTCCCTGCCTGTA | $(TG)_{11}$ | 428 | 51.74<br>54.05 | No hit |
| MF145 | F:TTGACTATTACGGTTTCAGG<br>R:GTTCTTTGATGTGAGGAATG | $(AC)_{12}$ | 360 | 52.95<br>52.98 | No hit |
| MF146 | F:TAGAGAGCAGTGATGGTAG<br>G<br>R:CAAAGGAAAGAAACACACA<br>C | $(GT)_{10}$ | 310 | 52.95<br>53.20 | No hit |
| MF147 | F:GTTTGTTCTGTGGCGTGTG<br>R:TAAGCGGTCTCTCTGTTTCC | $(GA)_{11}$ | 379 | 58.69<br>57.14 | PREDICTED: *Macaca fascicularis* smoothelin-like 2 (SMTNL2), transcript variant X2, mRNA |
| MF148 | F:TATTGGGTAGGGGAGTTTC<br>R:GGATTTTCAGATTAGGGAT<br>G | $(AC)_{14}$ | 495 | 54.04<br>53.72 | PREDICTED: *Macaca fascicularis* CTP synthase 2 (CTPS2), mRNA |
| MF149 | F:AATACCTGCCTTCCAAAGAG<br>R:ACAAGATTCCACAGTCCTTC | $(AC)_{13}$ | 373 | 56.05<br>54.09 | PREDICTED: *Macaca fascicularis* neuron navigator 1 (NAV1), transcript variant X9, mRNA |
| MF150 | F:AGGTAATAAGGACACTTGG<br>R:AGGCGTAACACAGAAATAC | $(TG)_{14}$ | 406 | 49.11<br>50.23 | No hit |
| MF151 | F:AAATGTTACCCTATGACTCC<br>R:AAGAAGAAATGACCCTCTG | $(CA)_{17}$ | 370 | 50.73<br>51.69 | No hit |
| MF152 | F:CAAAGAGAGGCTACAGAAG<br>R:TTAGATGAGGTGAGATGAA<br>G | $(CA)_{15}$ | 455 | 50.11<br>50.07 | PREDICTED: *Macaca fascicularis* apoptotic chromatin condensation inducer 1 (ACIN1), transcript variant X6, mRNA |
| MF153 | F:TTAGGTATGGTTTCTCCAC<br>R:GTTTCCCTTGACTCTGTC | $(GT)_{10}$ | 478 | 49.88<br>49.82 | No hit |
| MF154 | F:AGGCAATCCAACTAATCAA<br>C<br>R:GTTTCACCGTCCTTCTGTT | $(CA)_{11}$ | 378 | 54.30<br>55.08 | No hit |
| MF155 | F:ACTGATTCCCTTCTTTCC<br>R:AGAGTGCTTACAGATAATG<br>C | $(AC)_{11}$ | 283 | 51.34<br>49.66 | PREDICTED: *Macaca fascicularis* uncharacterized LOC102128257 (LOC102128257), transcript variant X1, ncRNA |
| MF156 | F:GCTTGTGCTGTTAGAAGGTG<br>R:TTCCCATCATTCACTCATTG | $(GT)_{12}$ | 368 | 56.17<br>56.85 | No hit |
| MF157 | F:CTCTCAGGAAGGGAAAGTG | $(AC)_{12}$ | 287 | 57.97 | No hit |

| | | | | | |
|---|---|---|---|---|---|
| | TC<br>R:TTATGGCAGTTGGATGTGCT | | | 59.15 | |
| MF158 | F:CGACAGGTGCTGAGAGAAA<br>R:TTGTTTGGAAGCCATTCAC | (AC)$_{10}$ | 465 | 57.59<br>57.07 | No hit |
| MF159 | F:CAGATGACTTTTGTAGCAGA<br>R:CTCCTTTGATGCGTAATG | (GT)$_{10}$ | 318 | 51.40<br>52.49 | No hit |
| MF160 | F:GGGAGACAGAAAGAGAATAC<br>R:CTACAAACTACGGTGAAATC | (TG)$_{10}$ | 260 | 50.38<br>49.93 | No hit |
| MF161 | F:TAGGTCTTCTCACCCACTT<br>R:CATCGTTTTACATTGACACC | (AG)$_{10}$ | 272 | 52.02<br>53.91 | No hit |
| MF162 | F:TTGGTGTTGTAAAGGAGAATGG<br>R:CTGTCAAGGGTCCAGAGGA | (AC)$_{15}$ | 461 | 58.99<br>58.73 | No hit |
| MF163 | F:GATGTTGCCCAGAAACTC<br>R:GGTATTTGCCTGAAGACG | (TG)$_{13}$ | 422 | 53.78<br>54.09 | PREDICTED: *Macaca fascicularis* TOR signaling pathway regulator (TIPRL), mRNA |
| MF164 | F:TGTCGTGAGCCAAAGATA<br>R:CGTGTGGTTGATACTCCA | (GT)$_{12}$ | 316 | 53.40<br>53.38 | No hit |
| MF165 | F:TGCTCAACACAAGAACTG<br>R:ACTGCTCATCTCATCTCAC | (GT)$_{18}$ | 489 | 50.96<br>50.44 | No hit |
| MF166 | F:CCTCCTTTACTTTCTGATTG<br>R:ATCCTTTCAACATCCTTCTC | (TC)$_{11}$ | 307 | 51.71<br>53.25 | PREDICTED: *Macaca fascicularis* uncharacterized LOC107128633 (LOC107128633), ncRNA |
| MF167 | F:TAGTGAAATGTGCCTGTAAC<br>R:TACCTGTGTGAGAGAACAAG | (TG)$_{10}$ | 430 | 51.19<br>50.98 | No hit |
| MF168 | F:TGGGCTCTCACAAATAATAG<br>R:ACAAAGTTCTGGTCTCTTCC | (AC)$_{14}$ | 347 | 53.02<br>53.38 | No hit |
| MF169 | F:ACACCACTCTACCCAGATG<br>R:CAGTTGCTCTTCATTTCTTG | (AC)$_{11}$ | 307 | 53.09<br>53.65 | No hit |
| MF170 | F:TCATCTGTTCTACCTTGTTC<br>R:ATAAATCCTGCTGAGACTG | (CT)$_{10}$ | 326 | 49.98<br>50.08 | No hit |
| MF171 | F:AGCCAATGTAACTGCCATA<br>R:AAAGGAGAGGAAAGTGAGGAG | (GT)$_{15}$ | 262 | 57.26<br>56.73 | No hit |
| MF172 | F:CACTAACATACCATCCAGAA<br>R:TCTACTTATCCCATCACTCTC | (CT)$_{10}$ | 382 | 50.90<br>50.93 | No hit |
| MF173 | F:GTCTGTTTATCCCATCTTG<br>R:ACTCCTTTACTCTCCCAAC | (GA)$_{14}$ | 567 | 50.18<br>50.66 | No hit |
| MF174 | F:GGGCTTCGTCTACTCCTTTC<br>R:AAATGGTTCCCGTCAGTCAC | (GT)$_{12}$ | 464 | 58.00<br>59.83 | No hit |
| MF175 | F:ATCCTCACATTTCTATGCTC<br>R:GGACATCAGTTTCTTCTGTA | (CA)$_{11}$ | 474 | 51.73<br>49.98 | No hit |
| MF176 | F:AGACCCCGCTTTCCACTAC<br>R:CAGCACAAGACTCCATCTCAA | (AC)$_{12}$ | 284 | 59.15<br>59.02 | No hit |
| MF177 | F:TAGAGCAGAGTAGATGGAAG<br>R:TTACAGCCTATGGTTTCAC | (TG)$_{12}$ | 260 | 49.68<br>51.05 | No hit |
| MF178 | F:ATGTAGGTTACTGGCAATC<br>R:TCTCTCTCCTTCTTCAATG | (GT)$_{11}$ | 394 | 49.89<br>50.02 | No hit |

| | | | | | |
|---|---|---|---|---|---|
| MF179 | F:AAAGAGAGGTTATGGAGGTA<br>R:TAGGAGTCACAAAGGGTTAC | $(AC)_{11}$ | 347 | 50.62<br>51.81 | No hit |
| MF180 | F:CTCTGGGCTTTGTTTATC<br>R:CACATACTGGGCATACTAAC | $(TG)_{10}$ | 397 | 50.57<br>50.60 | PREDICTED: *Macaca fascicularis* disintegrin and metalloproteinase domain-containing protein 1a-like (LOC102117847), mRNA |
| MF181 | F:CTCTGGGGAAAGAAAATG<br>R:TAACACATCATCACCTACCA | $(AC)_{14}$ | 290 | 53.06<br>52.05 | No hit |
| MF182 | F:GATACAACCTACAATCTTCTGG<br>R:TATGTCTACCCTGTGGAAAG | $(GT)_{12}$ | 253 | 53.15<br>52.68 | No hit |
| MF183 | F:CCTCAACTAAACACTCTGGT<br>R:CTTCGCTATCTACCTTTCAT | $(GT)_{20}$ | 250 | 51.80<br>51.48 | No hit |
| MF184 | F:GTTTACCATTTAGCCCTTC<br>R:CTGTCAAGATTTCAGGATG | $(TG)_{15}$ | 447 | 51.53<br>51.21 | No hit |
| MF185 | F:CTCTGTGGGACCTCTTCTTC<br>R:TCCGTTTGTATGAGTCTGTG | $(AC)_{11}$ | 457 | 56.41<br>54.56 | No hit |
| MF186 | F:TGTGTTTAGAGAAGACCATC<br>R:AGTCAGAGAGTCCCATTG | $(CA)_{11}$ | 367 | 49.98<br>50.25 | PREDICTED: *Macaca fascicularis* uncharacterized LOC107127258 (LOC107127258), transcript variant X2, ncRNA |
| MF187 | F:CCCAGGTTTATTTCCTATG<br>R:GAGAGAAGAGGGATGAGTAG | $(AC)_{14}$ | 325 | 52.21<br>50.43 | PREDICTED: *Macaca fascicularis* protocadherin beta 14 (PCDHB14), mRNA |
| MF188 | F:CACAGAAACAGAACAAATGG<br>R:TGAGTGCCTGAATAGAAGAA | $(GT)_{15}$ | 342 | 54.11<br>53.55 | No hit |
| MF189 | F:ATCAGAAGTTGAAGGTCAGT<br>R:CCCCTGGTAAATGAAATC | $(TAT)_{12}$ | 351 | 51.12<br>52.70 | PREDICTED: *Macaca fascicularis* uncharacterized LOC102119036 (LOC102119036), ncRNA |
| MF190 | F:TTTCCAGAGGAGGAGATG<br>R:GGCTAAGTCAAACAAACACA | $(GT)_{10}$ | 289 | 53.37<br>53.87 | No hit |
| MF191 | F:TGGACAAAATCAAGGCATAG<br>R:ATAGTCATCTCCCAGCACAC | $(TG)_{11}$ | 273 | 56.26<br>55.04 | No hit |
| MF192 | F:ACCTGAGTTGTTTCCAAG<br>R:ACACTGCCTACTTTACATTG | $(TG)_{12}$ | 368 | 50.59<br>50.40 | No hit |
| MF193 | F:TGTCTGAGGATGTCTCC<br>R:AGAATGAAGCACACTGAAC | $(CA)_{10}$ | 471 | 50.60<br>50.77 | No hit |
| MF194 | F:GCAGCAGTGAATAAAAGAAG<br>R:CTGAAACACACGAACTACAC | $(AT)_{10}$ | 324 | 52.97<br>51.43 | No hit |
| MF195 | F:CTGAGTGGAGTGTAGCATTG<br>R: GAGCCCAGCACATAGTAGG | $(TG)_{10}$ | 486 | 54.28<br>55.86 | No hit |
| MF196 | F:GTGAGGTGCTCTGAATCTGT<br>R:CTTGCTTTGTGCTTGATGTC | $(TG)_{13}$ | 332 | 55.25<br>57.05 | No hit |
| MF197 | F:CCTGACTACTATGGGTGAC<br>R:GCAACACTGAACCTAATCT | $(TG)_{11}$ | 414 | 49.97<br>49.99 | No hit |
| MF198 | F:CAGTTTTCTCCATTCCTAC<br>R:TCATTCTCTGACTACCTCTG | $(GT)_{16}$ | 268 | 49.53<br>50.02 | No hit |

| | | | | | |
|---|---|---|---|---|---|
| MF199 | F:ACACAACACAGCACACCTA<br>R:TGGAATCAACCTAAGCATC | $(TC)_{10}$ | 276 | 53.09<br>53.56 | No hit |
| MF200 | F:GTGGTAAACAGAATCCATA<br>G<br>R:TCTACAGGAAGGGTCTCTA | $(AC)_{10}$ | 437 | 49.60<br>49.56 | No hit |
| MF201 | F:ACGGTGAAGGAAGAGAAG<br>R:GCTGTTTGAAGTCCCTAC | $(AC)_{11}$ | 353 | 52.57<br>50.29 | No hit |
| MF202 | F:GTAGATGTTTGAGGTGTGTC<br>R:GGAAGTGTCTGTTGAATGA | $(TG)_{12}$ | 312 | 50.04<br>51.47 | No  hit |
| MF203 | F:TCTGTTCTGTGCCCTGTCAT<br>R:CCACTGCCTCCTTATTTCTT<br>T | $(TG)_{13}$ | 395 | 59.26<br>57.51 | No hit |
| MF204 | F:TCAGAACAGGGAGAGAGCA<br>R:CAGGATGGATGACAGCAG | $(GT)_{12}$ | 460 | 57.04<br>55.88 | PREDICTED: *Macaca fascicularis* uncharacterized LOC107131066 (LOC107131066), ncRNA |
| MF205 | F:CTCCTCAGAAACTTAGATGG<br>R:ACTGCTTACCTACCCTACAG | $(AC)_{12}$ | 343 | 51.58<br>51.26 | No hit |
| MF206 | F:CATTGGTGTTTCTGGGTTG<br>R:TTCGTGTTCTCTACATTCCA<br>TC | $(GT)_{15}$ | 310 | 57.33<br>56.82 | No hit |
| MF207 | F:ACCTTCCAATCTCTCTAAGC<br>R:GTATGCTGCGGTTACACTAT | $(AC)_{10}$ | 283 | 52.80<br>52.99 | No hit |
| MF208 | F:CTGTAGGATAGACCTGGAC<br>R:CCACGAAACATAAGGAAC | $(GT)_{14}$ | 345 | 49.16<br>51.18 | No hit |
| MF209 | F:GCCTGACACTTCCCATCAC<br>R:ATTTCATCCTGTGCTTTGGT | $(AC)_{16}$ | 318 | 59.04<br>57.11 | No hit |
| MF210 | F:CTGCTGTATGAACAAGACTG<br>R:CTGTAACCATCTCCATTTCT | $(GT)_{13}$ | 303 | 51.73<br>51.69 | No hit |
| MF211 | F:GTGTGTGTTGAAATCTGGTG<br>R:CTACATCCTCTACCTCCATT<br>G | $(TG)_{12}$ | 251 | 54.71<br>53.91 | No hit |
| MF212 | F:CTCTATTCTCATTCTCCTCA<br>R:AACTTTTGGGGTTCCTAC | $(CT)_{10}$ | 429 | 49.28<br>51.46 | No hit |
| MF213 | F:AACCTATTCCTCCCAAAATG<br>R:GGACTTGTTGACTATCTGTC<br>TTG | $(AC)_{10}$ | 400 | 55.66<br>55.65 | No hit |
| MF214 | F:TTCGTAACATCTCCTGACA<br>R:CACTTGCCTCCTCTCTATC | $(TTG)_{10}$ | 407 | 52.26<br>52.29 | No hit |
| MF215 | F:CACTATGGAATGTGTTTGTG<br>R:GTAACTGAGGTTTGGTTCAT | $(AC)_{13}$ | 323 | 52.17<br>51.60 | No hit |
| MF216 | F:GCAAACTGGACTTGTAAAT<br>G<br>R:CAACAGGAAAGAAAGAAG<br>AG | $(GT)_{11}$ | 250 | 53.39<br>51.84 | No hit |
| MF217 | F:GCTGAATGGGTAAATGAC<br>R:CTCCCTACAACCTCTTGA | $(GT)_{12}$ | 324 | 51.01<br>50.69 | No hit |
| MF218 | F:GTGTGAAGGGTCTCTAAAC<br>R:CTGCTGTGAACTAAGGAAC | $(TG)_{11}$ | 317 | 49.22<br>50.67 | No hit |
| MF219 | F:GTTCTCTTTGACATTTGACC<br>R:TTCTTCCTCCTTCTCTCTG | $(GT)_{10}$ | 325 | 52.08<br>51.93 | No hit |
| MF220 | F:CAAGAAGCAACACTACTCA<br>G<br>R:GGTGAAATCTGGTCAAAC | $(GT)_{13}$ | 384 | 51.05<br>50.72 | No hit |
| MF221 | F:GCAACGAGAGTGAAACTG | $(GT)_{17}$ | 467 | 52.43 | PREDICTED: *Macaca fascicularis* |

| | | | | | |
|---|---|---|---|---|---|
| | R:GGCGTAATCATACTTGGT | | | 50.73 | solute carrier family 26 (anion exchanger), member 4 (SLC26A4), transcript variant X3, mRNA |
| MF222 | F:TATGACCTACCTTTGCTATC<br>R:TACACAGAGATGAGAAGAC<br>C | $(GT)_{15}$ | 309 | 49.57<br>49.77 | No hit |
| MF223 | F:CTGTGCCTTACAAACAATAC<br>R:AGTGCTGAATGAGGTGAC | $(AC)_{10}$ | 388 | 50.87<br>51.32 | No hit |
| MF224 | F:AGTATCTGGCTTGCTGGTAA<br>R:CGCCTTCAGTTGTAATCTCT | $(AC)_{14}$ | 420 | 55.16<br>54.70 | No hit |
| MF225 | F:CTCCCTGTCTCCTTTATCAC<br>R:TTTCTTCCAGTTTCTGTTGG | $(AC)_{10}$ | 335 | 53.79<br>55.43 | No hit |
| MF226 | F:TCTTCCTTGCTTCTTTCTTG<br>R:GCTTGAGTTTGGTATTGAGT<br>AG | $(GT)_{10}$ | 340 | 54.97<br>53.57 | No hit |
| MF227 | F:GTCACCTTCTAATCTCTCAG<br>R:ATTCCCTCTCCAACTAAC | $(CT)_{10}$ | 434 | 48.68<br>48.62 | No hit |
| MF228 | F:CCCTTTTGGTCCTTTAGCC<br>R:CCTCTCCTCTCTCTCCCAGA | $(AC)_{12}$ | 339 | 58.63<br>58.64 | No hit |
| MF229 | F:CTGAAGACAGGAAAATCAC<br>R:GTCCCATAAACAGTGGTG | $(GA)_{14}$ | 479 | 50.28<br>51.66 | No hit |
| MF230 | F:GAGTTCTGCTTTGTTTTCC<br>R:CTCTTTCTGTCTCATAGGC | $(AC)_{11}$ | 466 | 52.44<br>49.73 | No hit |
| MF231 | F:TCAAAACTGATGTGGTGAC<br>G<br>R:TAAATGGGATGGCTCCTAA<br>G | $(TG)_{10}$ | 278 | 58.08<br>56.81 | No hit |
| MF232 | F:AAATCCAGACATAGCCTCA<br>G<br>R:TGTTTTCCTCTTCTCTCCTC | $(GT)_{12}$ | 290 | 54.44<br>53.61 | No hit |
| MF233 | F:CATTATCACTTCTGCCTTAC<br>R:ATCCTTCCTACTTCCAACT | $(GT)_{11}$ | 314 | 49.98<br>50.22 | No hit |
| MF234 | F:GTATCAGCGAGTTGTGTG<br>R:ACTGTGTCCTTGACTGGT | $(AC)_{11}$ | 414 | 50.25<br>50.47 | No hit |
| MF235 | F:ATACCACCACCACCATAC<br>R:ATACCTAAGAAACCCTAAG<br>C | $(AC)_{14}$ | 311 | 50.78<br>49.82 | No hit |
| MF236 | F:ATCAGTGGGTAGGTTGGAT<br>G<br>R:GAAGTCAGAAAGAAAGGAA<br>GC | $(TG)_{10}$ | 344 | 57.29<br>55.06 | No hit |
| MF237 | F:ACCTCCTGTTGGATTTAGAA<br>R:ACCCAGAACTTGCTACTGA<br>C | $(CT)_{11}$ | 454 | 53.85<br>54.40 | No hit |
| MF238 | F:AAACTTCCTCACACATTAGC<br>R:CAACCTTCTTCTATCAAACC | $(AC)_{16}$ | 386 | 51.97<br>51.50 | No hit |
| MF239 | F:AAAGAACCATCTACCAAAC<br>C<br>R:ATGAAAGCCATTGACACTA<br>C | $(TG)_{14}$ | 492 | 53.29<br>52.64 | No hit |
| MF240 | F:GTGACTCCTAACTATTGGCT<br>CTA<br>R:AAAGGTGAATGAAGCAGAA<br>G | $(AC)_{12}$ | 468 | 54.66<br>54.66 | No hit |
| MF241 | F:TATTCAAACCCTTACCCTTC | $(GTT)_{1}$ | 358 | 53.45 | No hit |

| | | | | | |
|---|---|---|---|---|---|
| | R:TTAGCCATTAGCCTTCATTC | $_{1}$ | | 54.75 | |
| MF242 | F:AACTGAAGGCAGGATTAG<br>R:ATTAGGCTGTGAAGTGTG | $(TG)_{11}$ | 272 | 50.07<br>48.85 | No hit |
| MF243 | F:GACACAACCAACAAATACTG<br>R:GAGTGAGTGGCTTACAAATG | $(TG)_{14}$ | 311 | 51.26<br>53.32 | No hit |
| MF244 | F:CTGTTTGCCATTTCTACTTG<br>R:GTGAGATTTACCACTCTGAAC | $(AC)_{12}$ | 494 | 53.59<br>51.28 | No hit |
| MF245 | F:TTCTTCATTCTGCTCTGTTG<br>R:GCCTATTCTACTCTTGTCATTC | $(AC)_{13}$ | 294 | 54.05<br>52.57 | No hit |
| MF246 | F:TGACAATCCTAACCCTCTTA<br>R:GTTCATCTCTCTCAGTTTGC | $(TA)_{10}$ | 393 | 52.36<br>52.34 | No hit |
| MF247 | F:AAATGCCAACCTGCCTAC<br>R:TTGTGAACCCATCCTCTG | $(AC)_{11}$ | 374 | 56.11<br>55.16 | No hit |
| MF248 | F:ATTCTCACCTGCTTCTTC<br>R:CTCAACCGTAACTGACTG | $(TG)_{11}$ | 416 | 49.78<br>49.35 | No hit |
| MF249 | F:ATCTTACAGGGTGTCTGAGG<br>R:ATTTCTCGGGTGTGTGTG | $(AC)_{10}$ | 370 | 54.14<br>55.09 | No hit |
| MF250 | F:CTCCATCTCGTTTCTTCTTC<br>R:GTGCCCAATACACACCTG | $(TG)_{12}$ | 337 | 54.10<br>55.61 | No hit |
| MF251 | F:GAATGTGTCAAGCACTCTC<br>R:AAACAGAACCAACAGGATAC | $(CT)_{15}$ | 291 | 51.12<br>51.60 | No hit |
| MF252 | F:TCAATCTCAAAGTAGTAGGC<br>R:CTGTTCTCTAAACCAAAGG | $(TG)_{11}$ | 398 | 49.65<br>49.90 | No hit |
| MF253 | F:CTGATGAACAAATGAGTGAC<br>R:GAGTTGAGGTAGAGAGGTTG | $(CAG)_{12}$ | 251 | 51.12<br>51.35 | PREDICTED: *Macaca fascicularis* spalt-like transcription factor 1 (SALL1), mRNA |
| MF254 | F:GTTTTGTTTTGTTCCTCTGC<br>R:GAATGAATGAATGGTCAGG | $(TG)_{12}$ | 450 | 54.99<br>53.59 | No hit |
| MF255 | F:TTCTGTGGCTTTGGTTTATG<br>R:TGTCAGGGATTGTGAGATTG | $(GT)_{12}$ | 350 | 56.34<br>56.97 | No hit |
| MF256 | F:AAGCATTCCTCTTAGCAC<br>R:CAAATCGCACAACATCTC | $(CA)_{10}$ | 319 | 50.24<br>53.18 | No hit |
| MF257 | F:CAAACACCCCACCAAGTA<br>R:TCCGCTCTACCTCTCTGTC | $(AG)_{10}$ | 274 | 55.12<br>55.52 | PREDICTED: *Macaca fascicularis* mitogen-activated protein kinase kinase 7 (MAP2K7), mRNA |
| MF258 | F:ACCCATTGCTTCCTCTCC<br>R:GGTGTTATTTGTGGTAGTTTGG | $(CT)_{18}$ | 273 | 57.55<br>56.17 | No hit |
| MF259 | F:TCATAGCAGAGAGTCCATT<br>R:ACCTCAAATACCCTTCATC | $(AC)_{10}$ | 325 | 50.40<br>51.35 | No hit |
| MF260 | F:GTCCAGAGACTAAGATACAT<br>R:GATACTCCTCCTTCACTAC | $(TG)_{14}$ | 327 | 45.30<br>45.48 | No hit |
| MF261 | F:CGCTTTTGTTTTGAGGAC<br>R:AGACTGAGAGTGGGAGGTG | $(TG)_{12}$ | 349 | 54.33<br>55.05 | No hit |
| MF262 | F:CCGAAGATAGTAATGATACC | $(GT)_{12}$ | 396 | 48.98<br>49.76 | No hit |

| | | | | | |
|---|---|---|---|---|---|
| | R:AAGGACTCAACAGAAAGG | | | | |
| MF263 | F:ATACTGGGAAGAAAGAAAGC<br>R:AACAACAACACATACCCTTG | $(GA)_{14}$ | 252 | 52.89<br>53.38 | No hit |
| MF264 | F:GGAAATGATAGGTGGAGAC<br>R:CACTGAGAAGCAAGAAGC | $(AC)_{15}$ | 332 | 51.69<br>51.77 | No hit |
| MF265 | F:ACTACTGCCCAACCCTATC<br>R:CCACGGTAACATTGACATT | $(CT)_{10}$ | 382 | 54.11<br>54.16 | No hit |
| MF266 | F:CTCAGTGGGTGGATTGACA<br>R:AGCGGGGTGTGTGTGTAT | $(AC)_{11}$ | 337 | 57.92<br>57.26 | No hit |
| MF267 | F:CAGTATGATTTCCCATTACC<br>R:AGTTCTGTTTCTCTGTTGTG | $(AAC)_{11}$ | 353 | 52.02<br>50.06 | No hit |
| MF268 | F:CTTTGTTCTGCCTTCCTC<br>R:GTGAAACCCCGTAAACTC | $(GT)_{11}$ | 392 | 53.25<br>52.80 | No hit |
| MF269 | F:GTGATAAGACAGGACAGAGG<br>R:ATAACTACTCCCATTCCAAC | $(TG)_{10}$ | 376 | 52.03<br>50.73 | No hit |
| MF270 | F:GTTTCTTGCTGATTTCTTCC<br>R:GTCCCACCACTTTGATTTAG | $(TG)_{11}$ | 441 | 54.13<br>54.62 | No hit |
| MF271 | F:CCAGGAGGAGACAACAGAG<br>R:CTGCCTATGGACTTGATGAG | $(GA)_{11}$ | 285 | 55.72<br>55.85 | No hit |
| MF272 | F:AGTGATAGTTTTACCCTTGG<br>R:TATGTTTGTGCTACCTTGAC | $(AC)_{16}$ | 257 | 51.06<br>51.19 | No hit |
| MF273 | F:TGGGGAGACAATAGAGAGTG<br>R:GATTCCAGGTCACAAAACTG | $(AC)_{16}$ | 277 | 55.20<br>55.55 | No hit |
| MF274 | F:ACTACAGAAGGAGCAGTGT<br>R:CTGATTGATGGAAGTGTTC | $(AC)_{10}$ | 264 | 49.41<br>50.97 | No hit |
| MF275 | F:GGTAACTCTTTGTTCTGTGTG<br>R:GTGATGTGTTCTATCCCTTC | $(AC)_{10}$ | 302 | 52.39<br>51.82 | No hit |
| MF276 | F:TTTCAAGGACATTTGGAGAG<br>R:TCTTAGCACCTGGCTTCC | $(CA)_{12}$ | 313 | 55.35<br>56.44 | No hit |
| MF277 | F:TAAAGAGTGTGATGGGAAG<br>R:AACAGCAGGTAACAGACAG | $(TC)_{10}$ | 449 | 50.87<br>50.99 | No hit |
| MF278 | F:TTTAGGTCTCCTCACTTAGC<br>R:GTCTTTCTATGCCACGATA | $(GT)_{18}$ | 431 | 51.41<br>51.12 | No hit |
| MF279 | F:AAGGATAGGAAGATGGTAAG<br>R:GAAAAGGGAAGAGAAAGTG | $(TC)_{10}$ | 262 | 50.21<br>51.50 | No hit |
| MF280 | F:AAAGAACCTCCAAGAGTATC<br>R:AGACACAGACAGACACAAAC | $(TG)_{21}$ | 444 | 50.08<br>50.46 | No hit |
| MF281 | F:GTGGGAAAACATAAAGGAC<br>R:GCAGTAAGCATTGAAAGATG | $(GT)_{18}$ | 299 | 51.94<br>53.64 | No hit |
| MF282 | F:CTTCTCTTGTCATAGTCACG<br>R:AAATCTGAGGTGTAATCCAG | $(CT)_{13}$ | 312 | 50.72<br>51.69 | No hit |
| MF283 | F:GGACACTTGGCAGAGATTA | $(GT)_{10}$ | 263 | 54.94 | No hit |

| | | | | | |
|---|---|---|---|---|---|
| | G<br>R:CCACTGTTTTCATTGTTGTG | | | 54.94 | |
| MF284 | F:CGATAGGATTCAGGTAAAGC<br>R:CACAGCAAACTCATACGG | $(TG)_{11}$ | 296 | 54.18<br>53.36 | PREDICTED: *Macaca fascicularis* phosphodiesterase 4D (PDE4D), transcript variant X13, mRNA |
| MF285 | F:GTGGATGGTATTCATTCTG<br>R:CACACTCACTCTGCTTCTTA | $(GT)_{11}$ | 462 | 50.85<br>51.38 | No hit |
| MF286 | F:CGATTTTACAGATGAGGAG<br>R:ACTAAGAGGTGTGGGTCTAC | $(GT)_{10}$ | 270 | 50.61<br>50.66 | PREDICTED: *Macaca fascicularis* uncharacterized LOC107130488 (LOC107130488), ncRNA |
| MF287 | F:ATGTCAGAGGAGTGTGAAT<br>R:GAGAGACCTAACTAATACAACC | $(TG)_{12}$ | 424 | 49.67<br>49.30 | No hit |
| MF288 | F:AGCGTTTGTGTGTGAAATG<br>R:AGCACCTCCTCCTTCTTAC | $(TG)_{11}$ | 336 | 55.57<br>53.48 | No hit |
| MF289 | F:TGTGACCTACTTTCAGTTTG<br>R:ATACAGTGGAGAGATACAACC | $(CA)_{11}$ | 489 | 51.14<br>50.70 | No hit |
| MF290 | F:CTCAGTGTAATGTGATTGAAGC<br>R:CTTGTGCTCTTTTCTTATGTCC | $(AG)_{13}$ | 345 | 55.07<br>55.86 | No hit |
| MF291 | F:TATTACACCACCCTTTCTC<br>R:GGTTTTCTTCCTGTGAAC | $(TC)_{11}$ | 272 | 49.88<br>50.03 | No hit |
| MF292 | F:AATGCTCCAAGAGGACAATG<br>R:GTTAGCCCAAGTCAAGATGG | $(AC)_{11}$ | 285 | 57.74<br>57.27 | No hit |
| MF293 | F:TTCCCATCCCCTTCTACTA<br>R:GGTATCCATTCTAACTTTGTCC | $(AC)_{14}$ | 258 | 55.18<br>54.49 | No hit |
| MF294 | F:TTTCTCTGCCCTATTGAAC<br>R:ACTCCCTGGTGGTTACTATC | $(GT)_{17}$ | 254 | 52.86<br>53.08 | No hit |
| MF295 | F:GGTTGATAGAATGTAGGACTC<br>R:GGGAAGAAGAAAGAGAATG | $(CA)_{13}$ | 321 | 50.42<br>51.38 | No hit |
| MF296 | F:GAGTTTCTGCCTCATTTC<br>R:TACCTTACCCTGTCTATTTG | $(TC)_{16}$ | 263 | 50.30<br>49.55 | No hit |
| MF297 | F:ATAGGTGTGTGTAAAGTCCAG<br>R:CATTCAGTGTGTTCATAAGC | $(AC)_{20}$ | 312 | 51.80<br>51.52 | No hit |
| MF298 | F:CCCGAGAGAGTAAATAACA<br>R:AGGAGGGTGTAGAAATGTAG | $(TG)_{13}$ | 375 | 50.30<br>50.91 | No hit |
| MF299 | F:ACCCAGATAACCTCTTTG<br>R:TAAAACCTGTGGCTTCTAC | $(AC)_{15}$ | 356 | 49.67<br>50.40 | No hit |
| MF300 | F:GTTGACATCTAAGGTTCTGC<br>R:ATGATTGAAAGAGCACACTC | $(AC)_{14}$ | 301 | 52.31<br>52.68 | No hit |

Table S2. Comparison of genetic diversity estimates for West Coast and East Coast Peninsular Malaysia populations of *M. fascicularis*.

| SSR locus | West Coast | | | East Coast | | |
|---|---|---|---|---|---|---|
| | Number of Alleles ($N_A$) | Observed Heterozygosity ($H_O$) | Expected Heterozygosity ($H_E$) | Number of Alleles ($N_A$) | Observed Heterozygosity ($H_O$) | Expected Heterozygosity ($H_E$) |
| MF016 | 2 | 0.200 | 0.200 | 1 | 0.000 | 0.000 |
| MF021 | 2 | 0.056 | 0.246 | 2 | 0.250 | 0.533 |
| MF029 | 4 | 0.000 | 0.629 | 2 | 0.000 | 0.400 |
| MF069 | 2 | 0.389 | 0.322 | 2 | 0.750 | 0.500 |
| MF080 | 2 | 1.000 | 0.514 | 2 | 1.000 | 0.533 |
| MF102 | 4 | 0.000 | 0.705 | 4 | 0.000 | 0.747 |
| MF113 | 4 | 0.235 | 0.604 | 4 | 0.625 | 0.792 |
| MF121 | 6 | 0.222 | 0.746 | 3 | 0.250 | 0.567 |
| MF130 | 2 | 0.133 | 0.460 | 2 | 0.125 | 0.125 |
| MF147 | 3 | 0.769 | 0.579 | 3 | 0.714 | 0.648 |
| MF188 | 2 | 0.000 | 0.315 | 3 | 0.125 | 0.542 |
| MF197 | 4 | 0.111 | 0.464 | 3 | 0.250 | 0.433 |
| MF225 | 2 | 0.333 | 0.286 | 2 | 0.125 | 0.125 |
| MF242 | 4 | 0.056 | 0.544 | 2 | 0.143 | 0.143 |
| MF255 | 5 | 0.500 | 0.614 | 5 | 0.250 | 0.758 |
| MF259 | 6 | 0.333 | 0.692 | 3 | 0.000 | 0.667 |
| MF261 | 3 | 0.111 | 0.527 | 2 | 0.000 | 0.533 |
| MF272 | 5 | 0.556 | 0.691 | 4 | 0.750 | 0.750 |
| MF273 | 1 | 0.000 | 0.000 | 2 | 0.000 | 0.400 |
| Mean | 3.316 | 0.263 | 0.481 | 2.684 | 0.282 | 0.484 |

Table S3. Overall genetic diversity estimates at each polymorphic SSR loci.

| SSR locus | Number of Alleles ($N_A$) | Allele Size Range (bp) | Observed Heterozygosity ($H_O$) | Expected Heterozygosity ($H_E$) | Fixation Index ($F_{ST}$) | Polymorphic Information Content (PIC) |
|---|---|---|---|---|---|---|
| MF016 | 2 | 350-375 | 0.125 | 0.125 | 0.053 | 0.110 |
| MF021 | 2 | 435-450 | 0.115 | 0.382 | 0.150 | 0.305 |
| MF029 | 4 | 350-385 | 0.000 | 0.691 | 0.249 | 0.625 |
| MF069 | 2 | 400-425 | 0.500 | 0.382 | 0.040 | 0.305 |
| MF080 | 2 | 285-315 | 1.000 | 0.510 | 0.000 | 0.375 |
| MF102 | 5 | 465-525 | 0.000 | 0.712 | 0.018 | 0.641 |
| MF113 | 4 | 400-450 | 0.360 | 0.675 | 0.042 | 0.613 |
| MF121 | 6 | 475-525 | 0.231 | 0.697 | 0.032 | 0.636 |
| MF130 | 2 | 440-450 | 0.130 | 0.372 | 0.116 | 0.298 |
| MF147 | 3 | 350-415 | 0.750 | 0.589 | 0.003 | 0.488 |
| MF188 | 3 | 445-465 | 0.042 | 0.393 | 0.033 | 0.325 |
| MF197 | 5 | 315-375 | 0.154 | 0.456 | 0.026 | 0.426 |
| MF225 | 2 | 325-375 | 0.269 | 0.238 | 0.027 | 0.206 |
| MF242 | 5 | 500-525 | 0.080 | 0.458 | 0.103 | 0.414 |
| MF255 | 5 | 335-400 | 0.423 | 0.651 | 0.011 | 0.595 |
| MF259 | 6 | 315-335 | 0.231 | 0.682 | 0.019 | 0.633 |
| MF261 | 3 | 265-285 | 0.077 | 0.528 | 0.021 | 0.418 |
| MF272 | 6 | 365-385 | 0.615 | 0.713 | 0.031 | 0.653 |
| MF273 | 2 | 435-450 | 0.000 | 0.145 | 0.143 | 0.132 |
| Mean | 3.630 | - | 0.269 | 0.495 | 0.059 | 0.431 |
